# Cardiometabolic Effects of Omnivorous vs Vegan Diets in Identical Twins: A Randomized Clinical Trial

**DOI:** 10.1001/jamanetworkopen.2023.44457

**Published:** 2023-11-30

**Authors:** Matthew J. Landry, Catherine P. Ward, Kristen M. Cunanan, Lindsay R. Durand, Dalia Perelman, Jennifer L. Robinson, Tayler Hennings, Linda Koh, Christopher Dant, Amanda Zeitlin, Emily R. Ebel, Erica D. Sonnenburg, Justin L. Sonnenburg, Christopher D. Gardner

**Affiliations:** 1 Stanford Prevention Research Center, Department of Medicine, School of Medicine, Stanford University, Palo Alto, California; 2 Department of Population Health and Disease Prevention, Program in Public Health, University of California, Irvine; 3 Quantitative Sciences Unit, Department of Medicine, Stanford University, Palo Alto, California; 4 Department of Microbiology and Immunology, School of Medicine, Stanford University, Stanford University, Palo Alto, California; 5 Chan Zuckerberg Biohub, San Francisco, California; 6 Center for Human Microbiome Studies, Stanford University School of Medicine, Stanford, California

## Abstract

**Importance:**

Increasing evidence suggests that, compared with an omnivorous diet, a vegan diet confers potential cardiovascular benefits from improved diet quality (ie, higher consumption of vegetables, legumes, fruits, whole grains, nuts, and seeds).

**Objective:**

To compare the effects of a healthy vegan vs healthy omnivorous diet on cardiometabolic measures during an 8-week intervention.

**Design, Setting, and Participants:**

This single-center, population-based randomized clinical trial of 22 pairs of twins (N = 44) randomized participants to a vegan or omnivorous diet (1 twin per diet). Participant enrollment began March 28, 2022, and continued through May 5, 2022. The date of final follow-up data collection was July 20, 2022. This 8-week, open-label, parallel, dietary randomized clinical trial compared the health impact of a vegan diet vs an omnivorous diet in identical twins. Primary analysis included all available data.

**Intervention:**

Twin pairs were randomized to follow a healthy vegan diet or a healthy omnivorous diet for 8 weeks. Diet-specific meals were provided via a meal delivery service from baseline through week 4, and from weeks 5 to 8 participants prepared their own diet-appropriate meals and snacks.

**Main Outcomes and Measures:**

The primary outcome was difference in low-density lipoprotein cholesterol concentration from baseline to end point (week 8). Secondary outcome measures were changes in cardiometabolic factors (plasma lipids, glucose, and insulin levels and serum trimethylamine *N*-oxide level), plasma vitamin B_12_ level, and body weight. Exploratory measures were adherence to study diets, ease or difficulty in following the diets, participant energy levels, and sense of well-being.

**Results:**

A total of 22 pairs (N = 44) of twins (34 [77.3%] female; mean [SD] age, 39.6 [12.7] years; mean [SD] body mass index, 25.9 [4.7]) were enrolled in the study. After 8 weeks, compared with twins randomized to an omnivorous diet, the twins randomized to the vegan diet experienced significant mean (SD) decreases in low-density lipoprotein cholesterol concentration (−13.9 [5.8] mg/dL; 95% CI, −25.3 to −2.4 mg/dL), fasting insulin level (−2.9 [1.3] μIU/mL; 95% CI, −5.3 to −0.4 μIU/mL), and body weight (−1.9 [0.7] kg; 95% CI, −3.3 to −0.6 kg).

**Conclusions and Relevance:**

In this randomized clinical trial of the cardiometabolic effects of omnivorous vs vegan diets in identical twins, the healthy vegan diet led to improved cardiometabolic outcomes compared with a healthy omnivorous diet. Clinicians can consider this dietary approach as a healthy alternative for their patients.

**Trial Registration:**

ClinicalTrials.gov Identifier: NCT05297825

## Key Points

**Question:**

What are the cardiometabolic effects of a healthy plant-based (vegan) vs a healthy omnivorous diet among identical twins during an 8-week intervention?

**Findings:**

In this randomized clinical trial of 22 healthy, adult, identical twin pairs, those consuming a healthy vegan diet showed significantly improved low-density lipoprotein cholesterol concentration, fasting insulin level, and weight loss compared with twins consuming a healthy omnivorous diet.

**Meaning:**

The findings from this trial suggest that a healthy plant-based diet offers a significant protective cardiometabolic advantage compared with a healthy omnivorous diet.

## Teaser

This randomized clinical trial of identical twin pairs compares the cardiometabolic effects of a healthy vegan diet with a healthy omnivorous diet.

## Introduction

Plant-based diets have gained recent popularity not only for their lower environmental impact compared with an omnivorous dietary pattern but also for their health benefits.^1,2^ The most significant global health crises affecting our generation are noncommunicable diseases and climate change, which are both inextricably linked to diet,^3^ and dietary patterns high in plants and low in animal foods can maximize health and environmental benefits.^4,5^ Plant-based diets contain a diverse family of dietary patterns, which encourage a reduced consumption of animal foods.^6^ Abundant evidence from observational and intervention studies^7,8,9,10,11,12,13^ indicates that vegan diets are associated with improved cardiovascular health and decreased risk of cardiovascular disease, likely because of the higher daily consumption of vegetables and fruits, legumes, whole grains and nuts, and seeds compared with other different types of dietary patterns.^14^

A vegan dietary pattern is typically lower in energy density but higher in fiber, vitamins, minerals, and phytonutrients compared with other dietary patterns.^15^ However, sometimes a vegan dietary pattern can limit specific nutrients, such as vitamin B_12_, iron, and calcium.^15,16^ Most studies^17,18^ examining vegan diets have been epidemiologic examinations, with a few reported clinical studies.^19,20^ A confounding factor to consider in epidemiologic studies is the bias of self-decided vegans who may differ from nonvegans in factors that may influence diet and health.^21^ In addition, a poorly formulated vegan diet can include low-quality plant foods, such as refined carbohydrates and added sugars.^22^ To address these concerns, we designed a trial to compare the cardiometabolic effects of a healthy vegan diet with a healthy omnivorous diet, exposing both groups to vegetables, legumes, fruits, whole grains, nuts, and seeds. To control for genetic differences that might alter the cardiometabolic effects of diet,^23^ we randomly assigned identical twins to follow the 2 diets for 8 weeks.

## Methods

This study followed the ethical standards of the Declaration of Helsinki^24^ and was approved by the Stanford University Human Subjects Committee on March 9, 2022. All study participants provided written informed consent. The trial protocol is given in Supplement 1. Additional methods are available in the eMethods in Supplement 2. This report follows the 25-item Consolidated Standards of Reporting Trials (CONSORT) reporting guideline of design, participants, interventions, outcomes, sample size, randomization, participant flow, baseline data, outcomes, ancillary analyses, limitations, and interpretation. Race and ethnicity data were collected via self-report and included to characterize the population for generalizability of findings.

### Study Design

This single-site, parallel-group, dietary intervention randomized clinical trial randomized healthy, adult identical twins to a healthy vegan or omnivorous diet for 8 weeks. Participant enrollment began March 28, 2022, and continued through May 5, 2022. The date of final follow-up data collection was July 20, 2022.

The primary outcome was the difference from baseline to 8 weeks in low-density lipoprotein cholesterol (LDL-C) levels between the diet groups. Secondary outcomes included differences from baseline to 8 weeks in body weight and levels of fasting triglycerides, high-density lipoprotein cholesterol, glucose, insulin, trimethylamine *N*-oxide (TMAO), and vitamin B_12_. Exploratory assessments included diet quality, adherence, and qualitative factors to help interpret the study’s findings (eFigure 1 in Supplement 2).

### Participants

We aimed to recruit 22 pairs of identical twins 18 years or older, a sample size determined by resource availability rather than a formal power calculation. Identical twins were recruited primarily from the Stanford Twin Registry and randomized using computerized random-number generation by a statistician (K.M.C.) blinded to the intervention, delivery, or data collection. Adult twins 18 years or older willing to consume a plant-based (vegan) or omnivore diet for 8 weeks were included. We excluded participants who weighed 45.36 kg (100 lb) or less, had a body mass index (calculated as weight in kilograms divided by height in meters squared) of 40 or higher, had an LDL-C level of 190 mg/dL or higher (to convert to millimoles per liter, multiply by 0.0259), had a systolic blood pressure of 160 mm Hg or higher or diastolic blood pressure of 90 mm Hg or higher, or were pregnant. Individuals self-reported race and ethnicity for the purpose of demographic reporting. Inclusion and exclusion criteria have been previously published.^25^

### Dietary Intervention

The study consisted of two 4-week phases: delivered meals and self-provided meals. Participants were provided all no-cost meals for the first 4 study weeks by a nationwide meal delivery company (Trifecta Nutrition). It was expected that after 4 weeks of food delivery and health educator counseling that participants would understand the amounts and types of foods they should purchase and prepare to achieve maximum adherence to the diets when self-providing meals.

Research staff worked with Trifecta Nutrition to develop menu offerings to match a healthy vegan and omnivorous diet, which emphasized vegetables, fruits, and whole grains while limiting added sugars and refined grains. During the initial 4 weeks, meals were delivered once each week, with 7 days of breakfast, lunch, and dinner meals. Participants also purchased and consumed snacks to meet their energy requirements following guidance from health educators.

Guiding principles were reinforced: (1) choose minimally processed foods; (2) build a balanced plate with vegetables, starch, protein, and healthy fats; (3) choose variety within each food group; and (4) individualize these guidelines to meet preferences and needs (eAppendix in Supplement 2). Although weight loss was not discouraged, our diet design did not include a prescribed energy restriction and was not intended to be a weight loss study. Participants were told to eat until they were satiated throughout the study.

### Collection of Dietary Intake

Two types of dietary data were collected. For the primary reporting data, 3 unannounced 24-hour dietary recalls—a structured interview intended to capture detailed information about food and drink intakes—were administered within a 1-week window (2 weekdays and 1 weekend day) of each time point (baseline, week 4, and week 8). Data were collected via telephone by a registered dietitian (L.R.D.) using Nutrition Data System for Research (Nutrition Coordinating Center). For the secondary reporting data, participants were encouraged to log their food intake using the Cronometer app (Cronometer Pro, Nutrition Tracking Software for Professionals; Cronometer); these data were used by health educators for real-time guidance of participants.

### Anthropometric and Metabolic Data

At 3 time points, participants visited the Stanford Clinical and Translational Research Unit after an overnight fast of 10 to 12 hours: baseline, 4 weeks (phase 1), and 8 weeks (phase 2). Blood draw and clinical measures were assessed using standard methods (eMethods in Supplement 2). Stool samples were collected for future analysis to examine changes to the gut microbiome (eg, microbial diversity), metabolites, inflammatory markers, and additional health factors.

### Statistical Analysis

Descriptive statistics, mean (SD) or number (percentage), were used for continuous and categorical variables, respectively. Table 1 presents baseline summary statistics by study group. For the primary analysis, we investigated differences between groups in the change from baseline to week 8 for LDL-C between vegan and omnivorous diets among identical twins. Primary analysis included all available data. A linear mixed model was used and included fixed effects for diet and time (baseline as reference) and an interaction effect for diet × time and a random effect for twin pair to account for the correlation between identical twins (ie, random intercept allowed intercept to vary for each twin pair). A Wald test was used to evaluate a significant difference in diet at 8 weeks from baseline (interaction term). Finally, we present model estimates (95% CIs) for diet at 8 weeks. For each secondary outcome, we evaluated a statistical model similar to the primary model as described herein.

**Table 1.** Baseline Characteristics of the Study Participants^a^

Characteristic	Vegan diet group (n = 22)	Omnivorous diet group (n = 22)	Combined groups (N = 44)
Sex			
Female	17 (77.3)	17 (77.3)	34 (77.3)
Male	5 (22.7)	5 (22.7)	10 (22.7)
Age, mean (SD), y	39.6 (12.7)	39.6 (12.7)	39.6 (12.7)
Highest level of education achieved (self-reported)			
High school graduate	0	2 (9.1)	2 (4.5)
Some college	7 (31.8)	5 (22.7)	12 (27.3)
College graduate	9 (40.9)	13 (59.1)	22 (50.0)
Some postgraduate school	2 (9.1)	0	2 (4.5)
Postgraduate degree	4 (18.2)	2 (9.1)	6 (13.6)
Race and ethnicity (self-reported)			
Asian	2 (9.1)	3 (13.6)	5 (11.4)
Black/African American	1 (4.5)	1 (4.5)	2 (4.5)
Native Hawaiian/Pacific Islander	1 (4.5)	0	1 (2.3)
White	16 (72.7)	16 (72.7)	32 (72.7)
Multiracial	2 (9.1)	2 (9.1)	4 (9.1)
Weight, mean (SD), kg			
Female	71.6 (12.9)	71.4 (12.1)	71.5 (12.5)
Male	68.7 (9.1)	72.7 (12.2)	70.7 (10.8)
Both sexes	70.9 (12.1)	71.7 (12.1)	71.3 (12.1)
BMI, mean (SD)			
Female	26.9 (5.0)	26.9 (4.9)	26.9 (4.9)
Male	22.6 (1.3)	23.0 (1.3)	22.8 (1.3)
Both sexes	25.9 (4.8)	26.0 (4.6)	25.9 (4.7)
Waist circumference, mean (SD), cm			
Female	86.3 (15.7)	87.3 (12.0)	86.8 (13.9)
Male	79.5 (7.3)	82.6 (8.4)	81.1 (7.9)
Both sexes	84.8 (14.5)	86.2 (11.4)	85.5 (13.0)
Blood pressure, mean (SD), mm Hg			
Systolic	120.7 (15.8)	127.1 (56.9)	123.9 (41.9)
Diastolic	74.7 (10.7)	75.0 (10.3)	74.9 (10.5)
Blood lipid level, mean (SD), mg/dL			
HDL-C	60.3 (12.8)	63.9 (15.0)	62.1 (13.9)
LDL-C	110.7 (32.0)	118.5 (35.2)	114.6 (33.5)
Triglycerides	101.8 (65.1)	106.1 (38.5)	104.0 (52.9)
Fasting glucose concentration, mean (SD), mg/dL	90.8 (9.4)	92.0 (9.6)	91.4 (9.4)
Fasting insulin level, mean (SD), μIU/mL	12.7 (4.6)	12.8 (5.7)	12.8 (5.1)
Vitamin B_12_ level, mean (SD), pg/mL	590 (468.4)	492 (184.5)	541 (355.3)

Abbreviations: BMI, body mass index (calculated as weight in kilograms divided by height in meters squared); HDL-C, high-density lipoprotein cholesterol; LDL-C, low-density lipoprotein cholesterol.

SI conversion factors: To convert HDL-C and LDL-C to mmol/L, multiply by 0.0259; triglycerides to mmol/L, multiply by 0.0113; glucose to mmol/L, multiply by 0.0555; insulin to pmol/L, multiply by 6.945; vitamin B_12_ to pmol/L, multiply by 0.7378.

^a^ Data are presented as number (percentage) of patients unless otherwise indicated.

Analyses were completed using R Studio, version 2022.12.0 (Posit Software). A 2-sided *P* ≤ .05 was considered to be statistically significant. No correction was applied for multiple comparisons, and secondary and exploratory analyses should be interpreted accordingly.

## Results

**Figure 1.**
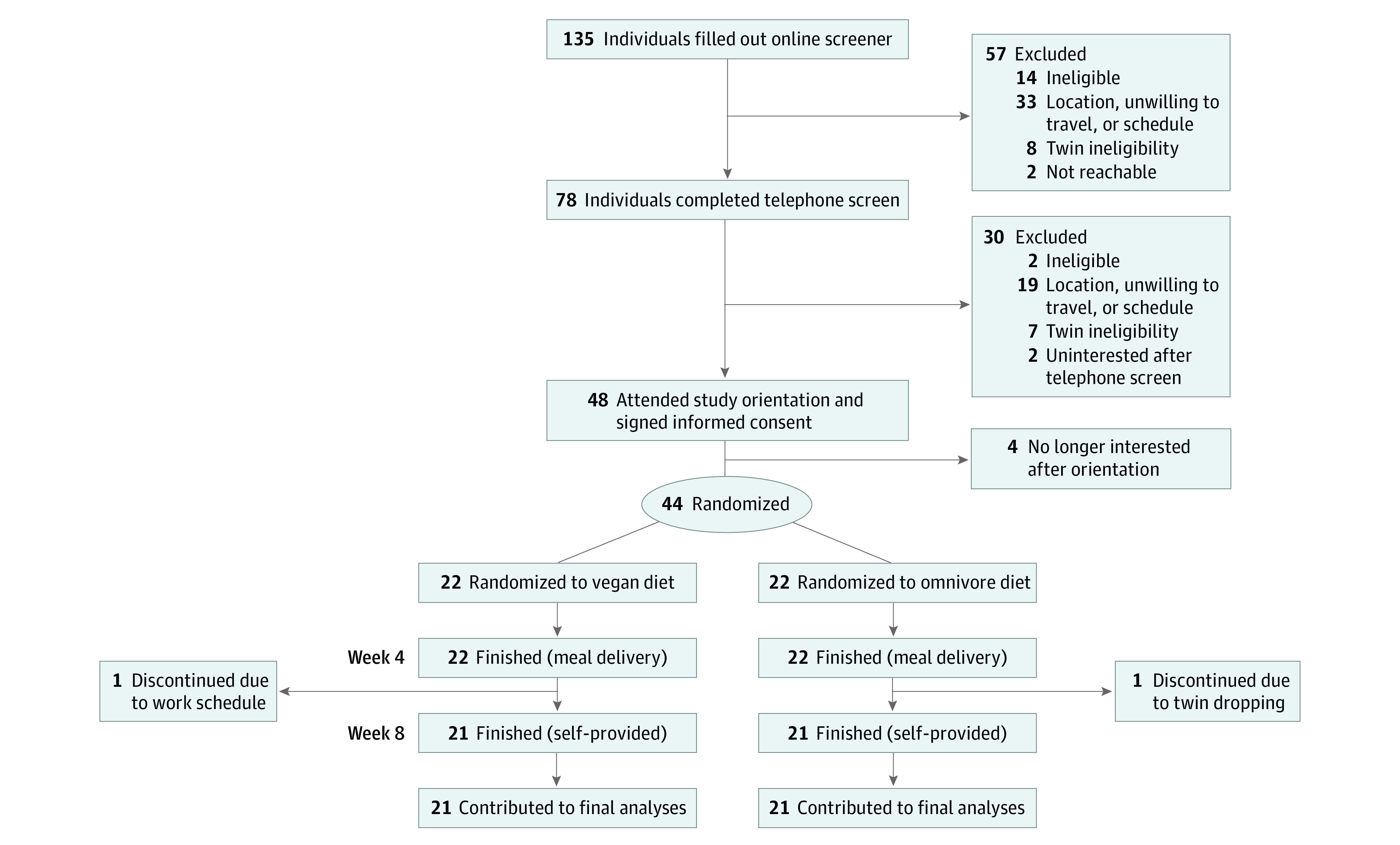
TwiNS CONSORT Flow Diagram

A total of 22 pairs of randomized twins (N = 44) were enrolled in the study. The CONSORT flow diagram of participants (Figure 1) shows 22 twin pairs randomized to receive either a vegan or omnivorous diet (1 twin per diet); 21 pairs in both groups contributed to the final analyses. Baseline characteristics (Table 1) included the following: mean (SD) age, 39.6 (12.7) years; 34 (77.3%) female and 10 (22.7%) male; 5 (11.4%) Asian, 2 (4.5%) Black/African American, 1 (2.3%) Native Hawaiian/Pacific Islander, 32 (72.7%) White, 4 (9.1%) multiracial, and mean (SD) body mass index, 26.9 (4.9). Most twins (33 of 42 [78.6%]) currently lived with their twin, and most reported being similar to their twin (29 of 42 [69.0%]) (Table 2; eTable 1 in Supplement 2).

**Table 2.** Cardiovascular Health Outcomes at the End of 8 Weeks and Main Effect Model Estimates for Primary and Secondary Outcome Analyses

Outcome^a^	Diet group, mean (SEM)^b^		Difference estimate (SE) [95% CI]^c^
	Vegan	Omnivorous	
Primary outcome			
LDL-C concentration, mg/dL	95.5 (6.3)	116.1 (6.7)	−13.9 (5.8) [−25.3 to −2.4]
Secondary outcomes			
HDL-C concentration, mg/dL	56.3 (2.8)	63.7 (4.0)	−3.6 (2.5) [−8.5 to 1.3]
Triglycerides level, mg/dL	93.5 (8.0)	98.2 (8.2)	−0.4 (14.7) [−28.9 to 28.1]
Vitamin B_12_ level, pg/mL	470.9 (53.1)	492.8 (37.3)	−103.0 (66.9) [−235.0 to 27.6]
TMAO level, μM	2.9 (0.3)	4.9 (1.1)	−2.1 (2.9) [−7.7 to 3.6]
Glucose concentration, mg/dL	90.2 (2.0)	91.6 (2.2)	−0.11 (2.3) [−4.6 to 4.4]
Insulin level, μIU/mL	10.5 (0.9)	13.7 (1.4)	−2.9 (1.3) [−5.3 to −0.4]
Weight, kg	69.5 (2.6)	71.7 (2.7)	−1.9 (0.7) [−3.3 to −0.6]

Abbreviations: HDL-C, high-density lipoprotein cholesterol; LDL-C, low-density lipoprotein cholesterol; TMAO, trimethylamine *N*-oxide.

SI conversion factors: To convert HDL-C and LDL-C to mmol/L, multiply by 0.0259; triglycerides to mmol/L, multiply by 0.0113; glucose to mmol/L, multiply by 0.0555; insulin to pmol/L, multiply by 6.945; vitamin B_12_ to pmol/L, multiply by 0.7378.

^a^ All laboratory data are fasting values from plasma (lipids, glucose, insulin, and vitamin B_12_) or serum (TMAO) specimens.

^b^ Means (SEMs) are unadjusted.

^c^ Primary and secondary outcomes fixed effects for diet and time (baseline as reference) and an interaction effect for diet (omnivore as reference) by time, a random effect for twin pair to account for the correlation between identical twins (ie, random intercept allowed intercept to vary for each twin pair), and a random effect for participant to account for correlation of longitudinal data.

### Diet and Nutrient Intake

Reported energy intake during each of the two 4-week phases (food delivery and self-provided) were lower compared with baseline for both groups (eFigures 1 to 5 and eTables 2 to 6 and 23 in Supplement 2). Intake of vegetables, animal-based protein sources, and plant-based protein sources by diet group and per intervention phase are provided in eFigures 6 to 12 and eTables 10 to 12 in Supplement 2. Additional results are available in the eResults, eTables 7 to 9, and eFigures 7 to 9 in Supplement 2.

### Primary Outcome

**Figure 2.**
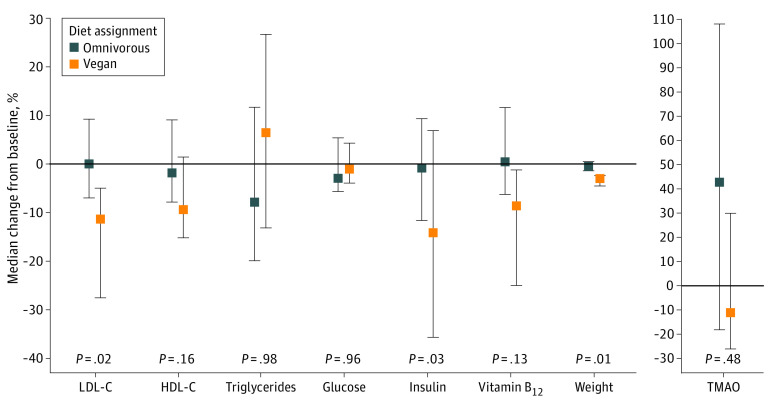
Median Change From Baseline to 8 Weeks in Primary and Secondary Outcomes Between Vegan and Omnivorous Diet Arms For primary and secondary outcomes, percent change and *P* values are presented. A Wald test was used to evaluate a significant difference in diet at 8 weeks from baseline (interaction term). Error bars indicate IQRs. HDL-C indicates high-density lipoprotein cholesterol; LDL-C, low-density lipoprotein cholesterol; and TMAO, trimethylamine *N*-oxide.

Participants receiving the vegan diet showed a mean (SD) decrease of 13.9 (5.8) mg/dL (95% CI, −25.3 to −2.4 mg/dL) in the unadjusted mean LDL-C level at 8 weeks from baseline compared with participants receiving the omnivorous diet (Table 2). As early as 4 weeks, we observed a significant decrease in mean LDL-C level among vegans compared with omnivores (eTable 20 in Supplement 2). The percentage of change from baseline to 8 weeks in primary and secondary outcomes between vegan and omnivorous diet groups (Figure 2) showed a significant decrease in LDL-C level among the vegan compared with the omnivore group (Table 2). Participants’ mean (SD) baseline LDL-C level was 114 (33.5) mg/dL,^26^ leaving minimal room for participants to improve through diet alone.

### Secondary Outcomes

Compared with participants receiving the omnivorous diet, participants receiving the vegan diet saw a significant mean (SD) decrease of 2.9 (1.3) μIU/mL in fasting insulin (95% CI, −5.3 to −0.4 μIU/mL) from baseline to 8 weeks (*P* = .03) (to convert to picomoles per liter, multiply by 6.945) (Table 2). Vegan participants had a significant mean (SD) decrease of −1.9 (0.7) kg in body weight (95% CI, −3.3 to −0.6 kg) from baseline to 8 weeks compared with participants on the omnivorous diet (*P* = .01) (Figure 2), although weight loss was observed for both diet groups. Vegans also experienced a larger but nonsignificant absolute median decrease in fasting high-density lipoprotein cholesterol, triglycerides, vitamin B_12_, glucose, and TMAO levels at 8 weeks from baseline compared with omnivores.

### Sensitivity Analysis

Three outlier TMAO levels greater than 15 μM were noted: 2 at baseline and 1 at 8 weeks. After the outliers were eliminated, the TMAO level was significantly different between diet groups at 8 weeks: in this analysis, participants on the vegan diet showed a mean (SD) decrease of −2.1 (0.7) μM (95% CI, −3.5 to −0.7 μM) in the difference of TMAO from baseline to 8 weeks compared with participants on the omnivorous diet (eFigure 13 in Supplement 2).

### Exploratory Analysis

Paired and unpaired 2-tailed *t* tests indicate minimal differences between statistical analysis approach (eTables 21 and 22 in Supplement 2). Participants receiving the omnivorous diet had nominally higher diet satisfaction at weeks 4 and 8 compared with vegan participants (eTable 13 in Supplement 2). Additional results are available in eResults and eTables 14 to 20 in Supplement 2.

## Discussion

In this randomized clinical trial of healthy, adult identical twins, the 8-week change in LDL-C level—the primary outcome—was significantly lower for twins receiving the vegan diet compared with twins receiving the omnivorous diet. Insulin levels and weight were also significantly lower among the twins on the vegan diet from baseline to 8 weeks. Vegan-diet participants had total lower protein intake as a percentage of calories, lower dietary satisfaction, lower intake of dietary cholesterol, but higher intake of vegetable servings and intake of dietary iron. Vegans had lower intake of vitamin B_12_, yet serum vitamin B_12_ levels were not statistically different than omnivores at 8 weeks, likely because of preserved stores.^27^ Long-term vegans are typically encouraged to take a cyanocobalamin (vitamin B_12_) supplement.

Two factors may have limited our opportunity to observe additional differences between the study groups. First, participants in both diet groups were assigned to eat a healthy diet, usually healthier compared with their prestudy dietary pattern demonstrated by increased vegetable intake and decreased refined grains intake. Even the omnivorous participants improved their diet quality during the 8-week intervention (eg, increased vegetables and whole grain intake and decreased added sugars and refined grains). Second, within both groups, potential differences in clinical end point changes may have been blunted because participants were healthy at baseline. For example, participants’ mean baseline LDL-C level was 114 mg/dL,^26^ leaving minimal room for participants to improve through diet alone. Nonetheless, we observed significant improvements in 3 clinical outcomes (LDL-C, insulin, and weight) among the vegan participants.

Our results corroborate a previous finding showing that eating a vegan diet can improve cardiovascular health.^28^ A larger body of evidence from randomized clinical trials suggests that vegetarian and other plant-based dietary patterns lower weight^29,30,31^ and improve lipid management,^30,32,33^ glucose metabolism,^33,34^ blood pressure,^35,36,37^ and cardiometabolic health.^38^ Our results also mirror a recently completed 2-year dietary intervention trial among African Americans randomized to a vegan or low-fat omnivorous diet, finding improvements in body weight and cardiovascular disease risk factors.^39^

Novel to this study was our population of identical twins, a valuable resource in scientific research that provided a unique opportunity to investigate the effects of a dietary intervention while controlling for genetic and environmental factors,^40^ influences that can significantly impact health outcomes, including body weight, cardiovascular health, and metabolic function.^40,41^ Because identical twins have nearly identical DNA and many shared experiences (eg, upbringing, geographic region growing up, and similar exposure to other variables), observed differences in health outcomes after adoption of different dietary patterns can largely be attributed to the diet itself.

We were surprised that TMAO concentrations did not significantly differ between diets at 8 weeks because of the higher meat content in the omnivorous diet and of the meat TMAO precursors choline and carnitine.^42,43^ Although some studies^44,45^ report a positive association between the concentration of serum TMAO and development of cardiovascular disease, whether TMAO is a bystander or mediator of disease remains unknown. In a sensitivity analysis that removed 3 TMAO outlier participants, lower TMAO levels were found in the vegan participants. Prior research^42,43^ has suggested that vegans have lower TMAO levels than meat or fish eaters because of the TMAO precursors choline and carnitine in animal products. In a recent crossover dietary trial (Study With Appetizing Plantfood-Meat Eating Alternative Trial [SWAP-MEAT]),^46^ participants consuming plant-based alternative meat vs animal meat had significantly lower TMAO concentrations. In addition to our 3 TMAO outliers, we observed variability among participants in TMAO concentration changes. Further investigation is needed on TMAO as a risk factor for cardiovascular disease and the association of dietary choline and carnitine vs fish with serum TMAO concentrations.

A recent meta-epidemiologic study^47^ examining dietary recommendations from current clinical practice guidelines recommends diets rich in unrefined plant foods and low in refined and animal-based foods. Clinical practice guidelines from the American Heart Association recommend that practitioners encourage patients to choose healthy sources of protein, mostly from plants, to promote cardiovascular health.^11,48^ Additionally, *Dietary Guidelines for Americans, 2020-2025*^49^ includes a healthy, vegetarian-style dietary pattern that can be adopted for improved health and chronic disease prevention. Although our findings suggest that vegan diets offer a protective cardiometabolic advantage compared with a healthy, omnivorous diet, excluding all meats and/or dairy products may not be necessary because research^22,50^ suggests that cardiovascular benefits can be achieved with modest reductions in animal foods and increases in healthy plant-based foods compared with typical diets. We believe lower dietary satisfaction in the vegan group may have been attributable to the strictness of the vegan diet, creating more barriers for people to follow the vegan diet guidelines. Some people may find a less restrictive diet preferable for LDL-C–lowering effects. Future studies assessing health benefits of less strict plant-based diets will be necessary to assess these benefits, especially in a study model limiting additional biases (eg, in twins). Within a clinical setting, patients should be supported in choosing a dietary pattern that fits their needs and preferences.^41,51^ Clinicians should allow patients to make informed choices that support them to choose which dietary approach is most suitable for them. At a population level, wider adoption of a culturally appropriate dietary pattern that is higher in plant foods and lower in animal foods can promote health and environmental benefits.^3,4,10,52^

### Strengths and Limitations

Several aspects of our design and implementation were strengths. First, enrolling identical twins was beneficial because we were able to eliminate the confounding influences of age, sex, and genetic factors that may affect clinical outcomes. Identical twins often share a similar environment and lifestyle, reducing environmental factors on the study results. Second, the initial 4-week period of food delivery facilitated participants’ high adherence to the diet, whereas the latter 4 weeks of self-provided foods increased generalizability. Third, we used LDL-C, a well-established cardiometabolic clinical value, as the primary outcome.^26^ Fourth, we assessed an extensive set of well-studied secondary clinical outcomes to evaluate overall cardiometabolic health. Fifth, diet data collection using the state-of-the-art Nutrition Data System for Research allowed us to assess and report on adherence—an important metric in free-living trials^53^—and compare macronutrient and micronutrient intakes. Sixth, previous trials^11,13,31,50,54,55^ have reported similar metabolic and weight loss benefits of vegan diets yet tended to focus on very low–fat vegan diets, study populations with diabetes or overweight, and comparison diets with limited attention to equipoise. Novelties of the current trial were the use of a more moderate- and higher-fat vegan diet (unsaturated fat),^11^ the generally healthy population without diabetes or overweight, and a healthy omnivorous comparison diet (eg, higher in vegetables and fiber than the baseline diet). Seventh, to provide fair and objective comparisons and avoid “straw man” comparators, we emphasized high-quality, exemplary dietary choices to participants on both diets.

The study also has some limitations. First, the adult twin population was generally healthy and may not be generalizable to other populations. Second, we studied a small sample size (N = 44); however, the use of monozygotic twins may reduce issues of reproducibility because the twins acted as their own controls. Third, study duration was short (8 weeks); however, in this study as well as several previous trials,^46,56^ clinically relevant changes in cardiovascular risk factors (eg, LDL-C and weight) were observed as early as 4 weeks into the intervention. Fourth, there was no follow-up period, which limited insights of poststudy stability and sustainability of diet behaviors. Fifth, our study was not designed to be isocaloric; thus, changes to LDL-C cannot be separated from weight loss observed in the study. We designed this study as a “free-living” study; thus, the behavior of following a vegan diet may induce the physiological changes we observed. However, the biological mechanisms cannot be determined to be causally from solely the vegan diet alone because of confounding variables (weight loss, decrease in caloric intake, and increase in vegetable intake). Sixth, diversity in education and socioeconomic status was lacking.

## Conclusions

In this randomized clinical trial, we observed cardiometabolic advantages for the healthy vegan vs the healthy omnivorous diet among healthy, adult identical twins. Clinicians may consider recommending plant-based diets to reduce cardiometabolic risk factors, as well as aligning with environmental benefits.

## Notes

Supplement 1.
Trial Protocol

Supplement 2. **eMethods.** Supplementary Methods **eResults.** Supplementary Results **eAppendix.** Plant-Based Dietary Intervention Reporting Checklist **eFigure 1.** TwiNS Study Design **eTable 1.** Twin Lifestyle Behaviors, n = 42 (21 Pairs of Twins) **eFigure 2.** Macronutrient Distribution by Phase and Diet **eTable 2.** Macronutrient Distribution by Phase and Diet **eFigure 3.** Fat Distribution by Phase and Diet **eTable 3.** Fat Distribution by Phase and Diet **eFigure 4.** Grain Distribution by Phase and Diet **eTable 4.** Grain Distribution by Phase and Diet **eFigure 5.** Protein Distribution by Phase and Diet **eTable 5.** Protein Distribution by Phase and Diet **eFigure 6.** Carbohydrate Distribution by Phase and Diet **eTable 6.** Carbohydrate Distribution by Phase and Diet **eFigure 7.** Dietary Cholesterol Distribution by Phase and Diet **eTable 7.** Dietary Cholesterol Distribution by Phase and Diet **eFigure 8.** Dietary Vitamin B12 Distribution by Phase and Diet **eTable 8.** Dietary Vitamin B12 Distribution by Phase and Diet **eFigure 9.** Dietary Iron Distribution by Phase and Diet **eTable 9.** Dietary Iron Distribution by Phase and Diet **eFigure 10.** Vegetable Servings Distribution by Phase and Diet **eTable 10.** Vegetable Servings Distribution by Phase and Diet **eFigure 11.** Animal-Based Protein Distribution by Phase and Diet **eTable 11.** Animal-Based Protein Distribution by Phase and Diet **eFigure 12.** Meat Alternatives Distribution by Phase and Diet **eTable 12.** Meat Alternatives Distribution by Phase and Diet **eTable 13.** Diet Satisfaction (D-Sat28) of Participants by Diet Assignment and Phase **eTable 14.** Self-Efficacy to Plan, Shop, Cook, and Choose Meals by Diet Assignment and Phase **eTable 15.** Diet Preferences of Participants by Diet Assignment **eTable 16.** Perceptions of Delivered, Pre-Prepared Meals by Diet Assignment **eTable 17.** Barriers to Adherence to Study Eating Patterns by Diet Assignment **eTable 18.** Factors that Improve Dietary Adherence and Sustainability by Diet Assignment **eTable 19.** Average Self-Rated Dietary Adherence by Diet Assignment and Phase **eTable 20.** Cardiovascular Health Outcomes at the End of 4 Weeks and Main Effect Model Estimates, Standard Errors, and 95% Confidence Interval for Primary and Secondary Outcome Analysis **eFigure 13.** Sensitivity Analysis of the Change in Trimethylamine N-Oxide (TMAO) With Three Outliers Removed, (Mean ± SE) **eTable 21.** Paired T-Tests of Cardiovascular Health Outcomes at the End of 8 Weeks **eTable 22.** Unpaired T-Tests of Cardiovascular Health Outcomes at the End of 8 Weeks **eTable 23.** Average Macronutrient and Micronutrient Composition of Trifecta Food Delivery Meals by Meal Type and Diet Assignment
eReferences

Supplement 3.
Data Sharing Statement
