# Error in Results Section

**DOI:** 10.1001/jamanetworkopen.2023.50422

**Published:** 2023-12-26

In the Original Investigation titled “Cardiometabolic Effects of Omnivorous vs Vegan Diets in Identical Twins: A Randomized Clinical Trial,”^1^ published on November 30, 2023, the last sentence in the Secondary Outcomes subsection of the Results incorrectly stated the secondary outcomes’ nonsignificant findings and did not correspond to the results correctly illustrated in Figure 2. This article has been corrected.
