# Supplementary material for: Cardiometabolic Effects of Omnivorous vs Vegan Diets in Identical Twins: A Randomized Clinical Trial
Source: JAMA Netw Open. 2023 Nov 30;6(11):e2344457. doi: 10.1001/jamanetworkopen.2023.44457 (PMC10690456; doi:10.1001/jamanetworkopen.2023.44457)
Supplement: Supplement 1. — Trial Protocol [file jamanetwopen-e2344457-s001.pdf]

Title : The Twins Nutrition Study (TwiNS): Vegan vs. Omnivore
Approval Period: 06/14/2022 - 12/31/2999

Modification Form ... 3
Personnel Info ... 3
Participant Population ... 6
Study Location ... 7
General Checklist ... 7
Funding ... 9
Resources ... 9
Expedited Category ... 11
Purpose ... 13
Radioisotopes or Radiation Machines ... 16
Drugs, Reagents, Chemicals, Devices ... 17
Medical Equipment for Human Subjects and Laboratory Animals ... 17
Participant Population(a-g) ... 17
Participant Population(h-m) ... 19
Risks(a-d) ... 20
Privacy And Confidentiality ... 22
Conflict Of Interest ... 26
Consent Background ... 26
Assent Background ... 27
Hipaa ... 28
Attachments ... 28

Obligations 30

Title : The Twins Nutrition Study (TwiNS): Vegan vs. Omnivore
Approval Period: 06/14/2022 - 12/31/2999

Modification

1. **Summarize your proposed changes.**

   -Adding a end-of-study questionnaire for participants

   -Adding two staff members - both are in the process of completing CITI Training and will not work on the protocol until that training is complete
   Elizabeth Lee - Health Educator
   Jordyn Van Hee - Diet Assessor

2. **Indicate Level of Risk**

   No Change

3. **Update the Conflict of Interest (COI) section if any changes in COI have been made since the last protocol submission.**

   N Is there a change in the conflicting interest status for any existing personnel on this protocol?

Protocol Director

Name		Degree (Program/year if student)		Position, e.g. Assistant Professor, Resident, etc.
Christopher D Gardner		PhD		Professor-Research
**Department**		**Phone**		**E-mail**
Medicine - Med/Stanford Prevention Research Center	5411	(650) 725-2751	(650) 725-6247	cgardner@stanford.edu
**CITI Training current**				Y

Admin Contact

Name		Degree (Program/year if student)		Position, e.g. Assistant Professor, Resident, etc.
Jennifer L. Robinson		PhD		Academic Prog Prof 2
**Department**		**Phone**		**E-mail**
Medicine - Med/Stanford Prevention Research Center	6151	(650) 736-8577		jlmorris@stanford.edu
**CITI Training current**				Y

Investigator

Name		Degree (Program/year if student)		Position, e.g. Assistant Professor, Resident, etc.
Justin L. Sonnenburg		PhD		Assoc Professor
**Department**		**Phone**		**E-mail**
Microbiology and Immunology	5124	(650) 721-2961	(650) 498-7147	Admin_Sonnenburg@stanford.edu
**CITI Training current**				Y

Title : The Twins Nutrition Study (TwiNS): Vegan vs. Omnivore
Approval Period: 06/14/2022 - 12/31/2999

Other Contact

**Name**		**Degree (Program/year if student)**		**Position, e.g. Assistant Professor, Resident, etc.**
Tayler Hennings		MPH		Study Coordinator
**Department** Medicine - Med/Stanford Prevention Research Center	5411	**Phone** (650) 723-8114		**E-mail** taylerkl@stanford.edu
**CITI Training current**				Y

Academic Sponsor

**Name**		**Degree (Program/year if student)**		**Position, e.g. Assistant Professor, Resident, etc.**
**Department**		**Phone**		**E-mail**
**CITI Training current**				

Other Personnel

**Name**		**Degree (Program/year if student)**		**Position, e.g. Assistant Professor, Resident, etc.**
Diane Demis				Asst Clinical Rsch Coord
**Department** Medicine - Med/Stanford Prevention Research Center	5537	**Phone** (650) 724-8310		**E-mail** ddemis@stanford.edu
**CITI Training current**				Y

**Name**		**Degree (Program/year if student)**		**Position, e.g. Assistant Professor, Resident, etc.**
Antonella Dewell				Casual - Non-Exempt
**Department** Medicine - Med/Stanford Prevention Research Center	5541	**Phone** (650) 736-8577	(650) 725-6906	**E-mail** adewell@stanford.edu
**CITI Training current**				Y

**Name**		**Degree (Program/year if student)**		**Position, e.g. Assistant Professor, Resident, etc.**
Dalia Perelman				Dietitian 2
**Department** Medicine - Med/Stanford Prevention Research Center	5541	**Phone** (650) 723-7296		**E-mail** daliap@stanford.edu

Title : The Twins Nutrition Study (TwiNS): Vegan vs. Omnivore
Approval Period: 06/14/2022 - 12/31/2999

**CITI Training current**				Y
**Name**<br>Matthew Landry		**Degree (Program/year if student)**		**Position, e.g. Assistant Professor, Resident, etc.**<br>Casual - Non-Exempt
**Department**<br>School of Medicine	5151	**Phone**		**E-mail**<br>mjlandry@stanford.edu
**CITI Training current**				Y
**Name**<br>Lindsay R Durand		**Degree (Program/year if student)**		**Position, e.g. Assistant Professor, Resident, etc.**<br>Diet Assessor/Research Assistant
**Department**<br>Medicine - Med/Stanford Prevention Research Center	5411	**Phone**<br>(650) 509-0007		**E-mail**<br>lrdurand@stanford.edu
**CITI Training current**				Y
**Name**<br>Erika Tribett		**Degree (Program/year if student)**		**Position, e.g. Assistant Professor, Resident, etc.**<br>Casual - Non-Exempt
**Department**<br>Medicine - Med/Stanford Prevention Research Center	2260	**Phone**		**E-mail**<br>etribett@stanford.edu
**CITI Training current**				Y
**Name**<br>Mandy Murphy Carroll		**Degree (Program/year if student)**		**Position, e.g. Assistant Professor, Resident, etc.**<br>Casual - Non-Exempt
**Department**<br>Medicine - Med/Stanford Prevention Research Center	5411	**Phone**<br>(650) 509-0004		**E-mail**<br>mandyiam@stanford.edu
**CITI Training current**				Y
**Name**<br>David Joel Maron		**Degree (Program/year if student)**<br>MD		**Position, e.g. Assistant Professor, Resident, etc.**<br>Professor-Univ Med Line
**Department**<br>Medicine - Med/Stanford Prevention Research Center	5406	**Phone**	(650) 725-1599	**E-mail**<br>david.maron@stanford.edu
**CITI Training current**				Y

Title : The Twins Nutrition Study (TwiNS): Vegan vs. Omnivore
Approval Period: 06/14/2022 - 12/31/2999

**Name**<br>Amanda Behrman Zeitlin		**Degree (Program/year if student)**		**Position, e.g. Assistant Professor, Resident, etc.**<br>Intern
**Department**<br>Medicine - Med/Stanford Prevention Research Center	7210	**Phone**		**E-mail**<br>abzeit@stanford.edu
**CITI Training current**				Y

**Name**<br>Cate Ward		**Degree (Program/year if student)**		**Position, e.g. Assistant Professor, Resident, etc.**<br>Temp - Postdoc
**Department**<br>Medicine - Med/Stanford Prevention Research Center	5541	**Phone**		**E-mail**<br>cateward@stanford.edu
**CITI Training current**				Y

**Name**<br>Elizabeth Lee		**Degree (Program/year if student)**		**Position, e.g. Assistant Professor, Resident, etc.**<br>Casual - Non-Exempt
**Department**<br>Medicine - Med/Stanford Prevention Research Center	5702	**Phone**		**E-mail**<br>elslee@stanford.edu
**CITI Training current**				Y

**Name**<br>Jordyn Danae Van Hee		**Degree (Program/year if student)**		**Position, e.g. Assistant Professor, Resident, etc.**
**Department**<br>Medicine - Med/Stanford Prevention Research Center		**Phone**		**E-mail**<br>jvanhee@stanford.edu
**CITI Training current**				N

Participant Population(s) Checklist — Yes/No

Participant Population(s) Checklist	Yes/No
• Children (under 18)	N
• Pregnant Women and Fetuses	N
• Neonates (0 - 28 days)	N
• Abortuses	N

Title : The Twins Nutrition Study (TwiNS): Vegan vs. Omnivore
Approval Period: 06/14/2022 - 12/31/2999

• Impaired Decision Making Capacity	N
• Cancer Subjects	N
• Laboratory Personnel	N
• Healthy Volunteers	Y
• Students	N
• Employees	N
• Prisoners	N
• Other (i.e., any population that is not specified above)	Y
• International Participants	N

Please enter the countries separated by comma

Study Location(s) Checklist

	Yes/No
• Stanford University	Y
• Clinical & Translational Research Unit (CTRU)	Y
• Stanford Hospital and Clinics	Y
• Lucile Packard Children's Hospital (LPCH)	
• VAPAHCS (Specify PI at VA)	
• Other (Click ADD to specify details)	

General Checklist

Multi-site

	Yes/No
• Is this a multi-site study? A multi-site study is generally a study that involves one or more medical or research institutions in which one site takes a lead role.(e.g., multi-site clinical trial)	N

Collaborating Institution(s)

	Yes/No
• Are there any collaborating institution(s)? A collaborating institution is generally an institution that collaborates equally on a research endeavor with one or more institutions.	N

Cancer Institute

	Yes/No
• Cancer-Related Studies (studies with cancer endpoints), Cancer Subjects (e.g., clinical trials, behavior/prevention) or Cancer Specimens (e.g., blood, tissue, cells, body fluids with a scientific hypothesis stated in the protocol).	N

Clinical Trials

	Yes/No
• Investigational drugs, biologics, reagents, or chemicals?	N

- Commercially available drugs, reagents, or other chemicals administered to subjects (even if they are not being studied)? N
- Investigational Device / Commercial Device used off-label? N
- IDE Exempt Device (Commercial Device used according to label, Investigational In Vitro Device or Assay, or Consumer Preference/Modifications/Combinations of Approved Devices) N

- Will this study be registered on clinicaltrials.gov? (See Stanford decision tree) Y
- Who will register for ClinicalTrials.gov? Y
  NCT# 05297825

Tissues and Specimens — Yes/No

- Human blood, cells, tissues, or body fluids (tissues)? Y
- Tissues to be stored for future research projects? Y
- Tissues to be sent out of this institution as part of a research agreement? For guidelines, please see https://sites.stanford.edu/ico/mtas https://sites.stanford.edu/ico/mtas Y

Biosafety (APB) — Yes/No

- Are you submitting a Human Gene Transfer investigation using a biological agent or recombinant DNA vector? If yes, please complete the Gene Transfer Protocol Application Supplemental Questions and upload in Attachments section. N
- Are you submitting a Human study using biohazardous/infectious agents? If yes, refer to the Administrative Panel on BioSafety website prior to performing studies. N
- Are you submitting a Human study using samples from subjects that are known or likely to contain biohazardous/infectious agents? If yes, refer to the Administrative Panel on BioSafety website prior to performing studies. N

Human Embryos or Stem Cells — Yes/No

- Human Embryos or Gametes? N
- Human Stem Cells (including hESC, iPSC, cancer stem cells, progenitor cells) N

Veterans Affairs (VA) — Yes/No

- The research recruits participants at the Veterans Affairs Palo Alto Health Care System(VAPAHCS). N
- The research involves the use of VAPAHCS non-public information to identify or contact human research participants or prospective subjects or to use such data for research purposes. N
- The research is sponsored (i.e., funded) by VAPAHCS. N
- The research is conducted by or under the direction of any employee or agent of VAPAHCS (full-time, part-time, intermittent, consultant, without compensation (WOC), N

on-station fee-basis, on-station contract, or on-station sharing agreement basis) in connection with her/his VAPAHCS responsibilities.

- The research is conducted using any property or facility of VAPAHCS. N

**Equipment**	**Yes/No**
• Use of Patient related equipment? If Yes, equipment must meet the standards established by Biomedical Engineering (BME) (650-725-5000)	N
• Medical equipment used for human patients/subjects also used on animals?	N
• Radioisotopes/radiation-producing machines, even if standard of care? ; More Info	N

**Payment**	**Yes/No**
• Subjects will be paid/reimbursed for participation? See payment considerations.	Y

**Funding**	**Yes/No**
• Training Grant?	N
• Program Project Grant?	N
• Federally Sponsored Project?	N
• Industry Sponsored Clinical Trial?	N

Funding

Funding - Grants/Contracts

Funding - Fellowships

Gift Funding

Name of Donor : Vogt Foundation

Dept. Funding

Other Funding

Resources :

a) Qualified staff.

Please state and justify the number and qualifications of your study staff.

Dr. Christopher Gardner, PhD, Principal Investigator of this project and Rehnborg Farquhar Professor of

Medicine at Stanford. He is experienced in overseeing studies of this type and subject, and has been conducting randomized controlled trials in free-living populations since 1993. Dr. Gardner has experience with multi- site study interventions as well as taste/food quality evaluations and consumer acceptance pilots. Most recently, he has been leading a study with 609 overweight and obese adults randomly assigned to 12 months of either a Very-Low-Fat or a Very-Low-Carbohydrate diet for weight loss (perhaps the largest, free-living, randomized, single-site, weight loss diet trial).

Dr. Jennifer Robinson, Ph.D., Gardner Research Group Associate Director and Project Data Manager. Dr. Robinson has many years of experience with all the administrative, regulatory, and scientific aspects of running research studies. For this study, she will be responsible for providing project staff with study protocol over-site.

Tayler Hennings, Study Coordinator. The Study Coordinator will be responsible for implementing all day-to-day operation for the study, including but not limited to the following: recruitment, participant management, database creation, data integrity.

Diane Demis, Research Assistant. The Research Assistant will be responsible for assisting the Study Coordinator, including but not limited to the following: developing research materials, screening participants, collecting data.

Dalia Perelman, RD, Dietitian and Lead Health Educator. The Lead Health Educator will be responsible for directing, managing and training staff related to all dietary intervention components of the study.

Cate Ward - Postdoctoral fellow

Our research team has substantial experience conducting similar clinical and community-based studies. In addition to the investigator(s) and staff listed above, we plan to recruit data collector(s) with appropriate experience in conducting research. All staff will be supervised by the protocol director, program manager or study coordinator and will complete training in research methodology and the use of human subjects in research.

b) Training.

Describe the training you will provide to ensure that all persons assisting with the research are informed about the protocol and their research-related duties and functions.

The Protocol Director and/or Program Manager will ensure that all staff are trained appropriately prior to the start of the protocol. This includes but is not limited to: HIPAA, CITI-GCP (GCP module required of all staff), study-specific protocol. This will be done via formal training to procedures at the beginning of the study and frequent face-to-face meetings, laboratory meetings, conference calls, direct supervision, observation and modeling. Our Program Manager has worked for a number of years in this capacity and is skilled at tracking and managing staff training records.

c) Facilities.

Provide the location(s) where the research will be conducted, including physical address if not conducted on site at Stanford University, Stanford Hospital on Pasteur Dr., Lucile Packard Children's Hospital on Welch Rd. or VAPAHCS. Describe the facilities and resources available to conduct the research at these sites.

The Stanford Prevention Research Center (SPRC) is housed in 20,000 sq. ft. located in the Stanford University Medical Center (SUMC) and at 1070 Arastradero Road in Palo Alto. Both spaces include offices for all faculty, staff, and administrative staff. The 1070 Arastradero location also has clinic space that will be used for participant screening and conference rooms that will be used for study-related meetings.

In addition, some visits will be conducted at the Clinical Translational Research Unit (CTRU), 800 Welch

Road. The CTRU nurses are highly skilled in assisting with the protocol procedures. Visits involving blood draws will be carried out there.

SPRC is on the SUMC network, which is compliant with HIPAA requirements and operates behind a firewall. Access to the Internet, electronic mail and secure file exchange is provided. SPRC investigators can access several secure servers and an array of statistical and other software provided by Stanford under site licenses; SPRC provides on-site computer support and daily back up.

Additionally, our coded samples will be sent out to multiple labs on an as needed basis for research analysis purposes only. An MTA will be obtained if necessary.

d) Sufficient time.

Explain the time that you and your research team will allocate to perform the research activities, including data analysis.

Our research group is very experienced in recruitment, screening and consenting eligible participants. In addition, we have a focused group to recruit from. Based on our prior experience and the proposed plan for this study, we feel confident that we have an ample amount of time to complete this project.

e) Access to target population.

Explain and justify whether you will have access to a population that will allow recruitment of the required number of participants.

We will be recruiting generally healthy, non-hospitalized, free-living, adults. For this study, we will be specifically recruiting twins, and we have access to recruit from several twin registries.

f) Access to resources if needed as a consequence of the research.

State whether you have medical or psychological resources available that participants might require as a consequence of the research when applicable. Please describe these resources.

We do not expect to need medical or psychological resources for this trial. If a participant does develop a side effect from the intervention, they will be instructed to contact any member of our research group. If a severe symptom of any kind, related or unrelated, is apparent, we will refer them to the emergency room immediately. However, it is more likely that someone will need to talk to our Health Educator (who is also a Registered Dietitian) for some dietary counseling. Outside of that expertise and assistance, we will not provide medical or psychological care ourselves, or provide financial assistance for care. We would simply refer them to their primary care physician for any physical or mental health concerns.

g) Lead Investigator or Coordinating Institution in Multi-site Study.

Please explain (i) your role in coordinating the studies, (ii) procedures for routine communication with other sites, (iii) documentation of routine communications with other sites, (iv) planned management of communication of adverse outcomes, unexpected problems involving risk to participants or others, protocol modifications or interim findings.

Expedited Form

A protocol must be no more than minimal risk (i.e., "not greater than those ordinarily encountered in daily life") AND must only involve human subjects in one or more of the following paragraphs.

Select one or more of the following paragraphs:

1. N **Clinical studies of drugs and medical devices only when condition (a) or (b) is met.**
   a) Research on drugs for which an investigational new drug application (21 CFR Part 312) is not required. (Note: Research on marketed drugs that significantly increases the risks or decreases the acceptability of the risks associated with the use of the product is not eligible for expedited review.)
   b) Research on medical devices for which
      i) an investigational device exemption application (21 CFR Part 812) is not required; or
      ii) the medical device is cleared/approved for marketing and the medical device is being used in accordance with its cleared/approved labeling.

2. Y **Collection of blood samples by finger stick, heel stick, ear stick, or venipuncture as follows:**
   a) from healthy, nonpregnant adults who weigh at least 110 pounds. For these subjects, the amounts drawn may not exceed 550 ml in an 8 week period and collection may not occur more frequently than 2 times per week; or
   b) from other adults and children, considering the age, weight, and health of the subjects, the collection procedure, the amount of blood to be collected, and the frequency with which it will be collected. For these subjects, the amount drawn may not exceed the lesser of 50 ml or 3 ml per kg in an 8 week period and collection may not occur more frequently than 2 times per week.

3. Y **Prospective collection of biological specimens for research purposes by non invasive means.**

4. Y **Collection of data through non invasive procedures (not involving general anesthesia or sedation) routinely employed in clinical practice, excluding procedures involving x-rays or microwaves. Where medical devices are employed, they must be cleared/approved for marketing. (Studies intended to evaluate the safety and effectiveness of the medical device are not generally eligible for expedited review, including studies of cleared medical devices for new indications.)**
   Examples:
   a) physical sensors that are applied either to the surface of the body or at a distance and do not involve input of significant amounts of energy into the subject or an invasion of the subject's privacy;
   b) weighing or testing sensory acuity;
   c) magnetic resonance imaging;
   d) electrocardiography, electroencephalography, thermography, detection of naturally occurring radioactivity, electroretinography, ultrasound, diagnostic infrared imaging, doppler blood flow, and echocardiography;
   e) moderate exercise, muscular strength testing, body composition assessment, and flexibility testing where appropriate given the age, weight, and health of the individual.

5. Y **Research involving materials (data, documents, records, or specimens) that have been collected, or will be collected solely for nonresearch purposes (such as medical treatment or diagnosis). (NOTE: Some research in this paragraph may be exempt from the HHS regulations for the protection of human subjects. 45 CFR 46.101(b)(4). This listing refers only to research that is not exempt.)**

6. N **Collection of data from voice, video, digital, or image recordings made for research purposes.**

7. Y **Research on individual or group characteristics or behavior(including, but not limited to, research on perception, cognition, motivation, identity, language, communication, cultural beliefs or practices, and social behavior) or research employing survey, interview, oral history, focus group, program evaluation, human factors evaluation, or quality assurance methodologies. (NOTE: Some research in this category may be exempt from the HHS regulations for the protection of human subjects. 45 CFR 46.101(b)(2) and (b)(3). This listing refers only to research that is not exempt.)**

Title : The Twins Nutrition Study (TwiNS): Vegan vs. Omnivore
Approval Period: 06/14/2022 - 12/31/2999

1. Purpose

a) In layperson's language state the purpose of the study in 3-5 sentences.

A vegan diet has been promoted as having a much lower environmental impact, but there is some controversy about the health effects of following a vegan diet. Some believe that this plant-based diet can help prevent many of the chronic diseases that affect us, such as heart disease, cancer, and Alzheimer's disease and would also help in managing body weight. Others claim that the vegan diet does not provide all the required nutrients, such as adequate amounts of protein, vitamin B12 and the minerals iron and calcium, and thus it would not promote optimal health. This study is designed to investigate the health impact of a vegan diet compared to a usual, omnivorous diet. We plan to study these diets in twins, where one twin follows a vegan diet and the other twin follows an omnivorous diet, thus we control for genetic differences that might impact the effect of the diet.

b) State what the Investigator(s) hope to learn from the study. Include an assessment of the importance of this new knowledge.

The vegan diet is exclusively restricted to the consumption of plant-based foods and is becoming increasingly popular among the population of the Western world, both for its lesser environmental impact, animal rights interest, and for its purported benefits on health. Many individuals are interested in following such a diet but are concerned about getting all the required nutrients in adequate amounts. Some of the nutrients of concern are protein and vitamin B12, and people believe they might not be able to perform at the same level and maintain their energy levels through the day. During this study, we will evaluate the nutrient intake in both the vegan and the omnivorous diet. We will also measure physiologic markers of health such as lipid levels, HbA1C, heart rate, and weight, and we will also look at the effect of the diets on the microbiota. In addition to measuring the effect of the diet, we will monitor adherence to the diet, and survey participants on the ease/difficulty in following a vegan diet as well as their energy levels and sense of wellbeing.
Thus, this study will help us better understand the health impact and feasibility of following a vegan diet. These results will be of much interest to the general public and the health care professionals.

c) Explain why human subjects must be used for this project. (i.e. purpose of study is to test efficacy of investigational device in individuals with specific condition; purpose of study is to examine specific behavioral traits in humans in classroom or other environment)

The purpose of this study is to assess how the cardiovascular health, gut microbiome, and metabolic status of humans can be affected by consuming exclusively plant-based foods.

2. Study Procedures

a) Please SUMMARIZE the research procedures, screening through closeout, which the research participant will undergo. Sections in the protocol attached in section 16 can be referenced, BUT do not copy the clinical protocol. Be clear on what is to be done for research and what is part of standard of care. For research involving collaborators, please specify the respective roles of Stanford and each collaborator on the protocol.

RECRUITMENT/SCREENING
Eligibility is first determined by having participants complete an online screening questionnaire. Those potential subjects who meet the initial eligibility criteria will be invited to an orientation meeting (via zoom).

ORIENTATION
If deemed eligible at the time of the in-person screening clinic visit, participants will participate in an orientation where study details are described and questions answered. This is where participants will be consented.

RANDOMIZATION
Participants will not be randomized until all baseline clinic visits, food logs, and online questionnaires are completed. One of the individuals in each pair of twins will be randomized to a vegan diet, and the other will be randomized to an omnivore diet.

DATA COLLECTION
We will collect stool, blood (through venous blood draw at the CTRU), and blood microsamples using a Tasso device at home, food logs, psychosocial data, cognitive data, and physical activity data from all participants at the following time points:

Week -1
Week 0 (Baseline and time of randomization, CTRU)
Week 2
Week 4 (CTRU)
Week 6
Week 8 (end of intervention, CTRU)

INTERVENTION
The individuals randomized to a vegan diet will be counseled to consume exclusively plant-based foods that are minimally processed (i.e., vegetables, fruits, beans, nuts, seeds).
The individuals randomized to an omnivore diet, will be counseled to consume a minimally processed diet that includes animal foods, such as meats, dairy, and egg in addition to the plant-based foods.
All participants will receive dinners and lunches for the first 4 weeks of the diet intervention from a food delivery company (Trifecta).
Participants will make their own meals during the last 4 weeks of the diet intervention. They will be followed up by study health educators to achieve high adherence to the diets.

MEASUREMENTS
a. Clinical measurements (e.g.: height, weight, waist circumference, blood pressure)
b. Blood samples (e.g.: lipids, glucose, insulin, immune markers)
c. Stool samples (e.g.: 16S)
d. Dietary intake: food logs collected via Cronometer (a food logging phone and web application) and text message questionnaires collected via Twilio, a service that integrates directly with REDCap. During weeks 0, 4, and 8 dietary intake will be assessed with unannounced 24-hour
diet recalls using Nutrition Data System for Research (NDSR).

e. Questionnaires online questionnaires collected via REDCap (e.g.: Food intake / satisfaction, gastrointestinal, psychosocial, and physical activity)
f. Cognitive data (via Cambridge Cognition CANTAB)

b) Explain how the above research procedures are the least risky that can be performed consistent with sound research design.

All assessments will be conducted by trained, professional individuals.
None of the procedures are invasive. The blood sampling will be performed by trained nurses at the CTRU of Stanford Hospital.
The intervention is a dietary intervention with what is evidenced to be health promoting diets.
In summary, all of the procedures involved in this study are low-risk procedures.

c) State if deception will be used. If so, provide the rationale and describe debriefing procedures. Since you will not be fully informing the participant in your consent process and form, complete an alteration of consent (in section 13). Submit a debriefing script (in section 16).

No deception will be used.

d) State if audio or video recording will occur. Describe what will become of the recording after use, e.g., shown at scientific meetings, erased. Describe the final disposition of the recordings.

Parts of the study will be video recorded. The final product will become part of a Netflix documentary on vegan diets. Only a select number of twin pairs will be selected to participate in the full Netflix documentary. Others may be filmed during orientation or our results presentation. Netflix will provide their own release forms for participants to consent to being filmed. The filming will not be used for research purposes. Participants will also have the option to opt-out of filming, which will not effect their research participation whatsoever.

e) Describe alternative procedures or courses of treatment, if any, that might be advantageous to the participant. Describe potential risks and benefits associated with these. Any standard treatment that is being withheld must be disclosed in the consent process and form. (i.e. standard-of-care drug, different interventional procedure, no procedure or treatment, palliative care, other research studies).

We are recruiting healthy participants to this study. Their alternative is not to join the study. No benefits/risks in not joining the study.

f) Will it be possible to continue the more (most) appropriate therapy for the participant(s) after the conclusion of the study?

Yes, participants will be able to choose which diet they would like to continue to consume at the end of the intervention: either a vegan or an omnivore diet.

g) Study Endpoint. What are the guidelines or end points by which you can evaluate the different treatments (i.e. study drug, device, procedure) during the study? If one proves to be clearly more effective than another (or others) during the course of a study, will the study be terminated before the projected total participant population has been enrolled? When will the study end if no important differences are detected?

There is no plan for early termination or interim data evaluation.

3. Background

a) Describe past experimental and/or clinical findings leading to the formulation of the study.

Several studies have shown the beneficial effects of a vegan diet on human health due to the higher daily consumption of vegetables, whole grains nuts, legumes and seeds, suggesting that vegan individuals have healthier lifestyle behaviors, compared to individuals following different types of diet. Such health benefits have been suggested to include, among others, a lower incidence of non-communicable diseases (NCD), such as cardiovascular disease, colon cancer, type 2 diabetes, liver disease, as well as better weight management.
On the other hand, less is known about whether any negative health implications could result from nutrient deficiencies arising from a vegan diet. Studies suggest that subjects following this type of diet are more likely to show deficiencies in macro-as well as micronutrients and there is debate on whether a vegan diet should be supplemented with various nutrients, including iron, zinc, iodine, selenium, calcium, long-chain n-3-fatty acids, vitamin B12, and vitamin D.
Most of the studies have been epidemiological studies, and very few clinical studies have been done to answer these questions.
In addition, no studies have been done to study the effect of a vegan diet on the microbiota composition and how that might differ from the microbiota in an omnivorous diet. Since we now know of the large impact of the microbiota on human physiology, this question is of great interest.

b) Describe any animal experimentation and findings leading to the formulation of the study.

Not applicable.

4. Radioisotopes or Radiation Machines

a) List all standard of care procedures using ionizing radiation (radiation dose received by a subject that is considered part of their normal medical care). List all research procedures using ionizing radiation (procedures performed due to participation in this study that is not considered part of their normal medical care). List each potential procedure in the sequence that it would normally occur during the entire study. More Info

Identify Week/Month of study	Name of Exam	Identify if SOC or Research

b) For research radioisotope projects, provide the following radiation-related information:

Identify the radionuclide(s) and chemical form(s).

For the typical subject, provide the total number of times the radioisotope and activity will be administered (mCi) and the route of administration.

If not FDA approved provide dosimetry information and reference the source documents (package insert, MIRD calculation, peer reviewed literature).

c) For research radiation machine projects, provide the following diagnostic procedures:

For well-established radiographic procedures describe the exam.

For the typical subject, identify the total number of times each will be performed on a single research subject.

For each radiographic procedure, provide the setup and technique sufficient to permit research subject dose modeling. The chief technologist can usually provide this information.

For radiographic procedures not well-established, provide FDA status of the machine, and information sufficient to permit research subject dose modeling.

d) **For research radiation machine projects, provide the following therapeutic procedures:**

For a well-established therapeutic procedure, identify the area treated, dose per fraction and number of fractions. State whether the therapeutic procedure is being performed as a normal part of clinical management for the research participants's medical condition or whether it is being performed because the research participant is participating in this project.

For a therapeutic procedure that is not well-established, provide FDA status of the machine, basis for dosimetry, area treated, dose per fraction and number of fractions.

5. Devices

a) **Please list in the table below all Investigational Devices (including Commercial Devices used off-label) to be used on participants**

b) **Please list in the table below all IDE Exempt Devices (Commercial Device used according to label, Investigational In Vitro Device or Assay, or Consumer Preference/Modifications/Combinations of Approved Devices) to be used on participants.**

6. Drugs, Reagents, or Chemicals and Devices

a) **Please list in the table below all investigational drugs, reagents or chemicals to be administered to participants.**

b) **Please list in the table below all commercial drugs, reagents or chemicals to be administered to participants.**

7. Medical Equipment for Human Subjects and Laboratory Animals

If medical equipment used for human patients/participants is also used on animals, describe such equipment and disinfection procedures.

Not applicable.

8. Participant Population

a) **State the following: (i) the number of participants expected to be enrolled at Stanford-affiliated site(s); (ii)the total number of participants expected to enroll at all sites; (iii) the type of participants (i.e. students, patients with certain cancer, patients with certain cardiac condition) and the reasons for using such participants.**

(i) 48 individuals, 24 pairs of twins

(ii) Healthy adult population

b) State the age range, gender, and ethnic background of the participant population being recruited.

Age Range: 18+ years old

Gender: both genders will be included

Ethnicity: all ethnicities

c) State the number and rationale for involvement of potentially vulnerable subjects in the study (including children, pregnant women, economically and educationally disadvantaged, decisionally impaired, homeless people, employees and students). Specify the measures being taken to minimize the risks and the chance of harm to the potentially vulnerable subjects and the additional safeguards that have been included in the protocol to protect their rights and welfare.

No vulnerable populations will be involved in this study.

d) If women, minorities, "https://stanfordmedicine.box.com/shared/static/4aj1fth309551do70wg6t06pukl98as6.pdf" target="_blank"non-English speaking individuals, or children are not included, a clear compelling rationale must be provided (e.g., disease does not occur in children, drug or device would interfere with normal growth and development, etc.).

Women and minorities will be included in this study. Children will not be included as this study is designed to look at a healthy adult population.

e) State the number, if any, of participants who are laboratory personnel, employees, and/or students. They should render the same written informed consent. If payment is allowed, they should also receive it. Please see Stanford University policy.

Laboratory personnel, employees and students are not specifically targeted for recruitment, but they will have the same right to participate as the general public.

f) State the number, if any, of participants who are healthy volunteers. Provide rationale for the inclusion of healthy volunteers in this study. Specify any risks to which participants may possibly be exposed. Specify the measures being taken to minimize the risks and the chance of harm to the volunteers and the additional safeguards that have been included in the protocol to protect their rights and welfare.

All participants in the study will be generally healthy volunteers. The rationale is that these individuals may still benefit from lifestyle and behavioral interventions to further improve their health.

Measures will be taken to minimize risk and to protect participants' rights and welfare.

g) Describe your plan to identify and recruit potential participants including who will inform them about the study and how they will be initially contacted by the researchers (e.g., https://med.stanford.edu/spectrum/researcher-resources/participant-engagement.html Research Engagement services; chart review; treating physician; ads including social media posts). All final or revised recruitment materials must be approved by the IRB before use. Contacting potential participants is not permitted prior to IRB approval. See https://stanfordmedicine.box.com/shared/static/8uebsdjrrqjyauanjp9i9d0gm1i480co.pdf Recruitment Guidance for additional information.

We will recruit participants primarily from the Stanford Twin Registry, which is a list of twin pairs that have indicated interest in participating in Stanford research studies. We also have the option to recruit participants outside of the Bay Area, in which we may use other twin registries (i.e. Michigan State Twin Registry).

Additionally, Netflix, Inc. will be pre-recruiting potential twins that they are interested in filming. The group responsible for the filming is simply sending out a casting call to various twin registries to get initial interest from twins interested in being filmed and being part of a dietary research study. They will not

present any details of the study, but will speak generally about the documentary and find twins that will be interested in being in a documentary about vegan diets to set us up with a pipeline of potential participants.

We may also: post the information on our website and encourage participants to share information about the study with their friends; use paid radio, music streaming service (Pandora, Spotify, etc.) and newspaper (both online and in print) advertising; post paper flyers, and email participants on the Stanford WELL for Life Registry.

h) Inclusion and Exclusion Criteria.

Identify inclusion criteria.

- Age 18+

- 1/2 of a pair of twins that will both be participating

- Wiling to consume a plant-based diet (vegetables, fruit, whole grains, legumes, etc.)

- Willing to consume meat/eggs (beef, pork/sausage, chicken, eggs) >= 1 time a day

- Willing to consume dairy (milk, yogurt, cheese) >= 1 time a day

Identify exclusion criteria.

- Weight < 110 lb

- BMI >= 40

- LDL-C > 190 mg/dL

- Systolic Blood Pressure > 160 mmHg OR Diastolic blood pressure > 90 mmHg

- Pregnant, lactating or planning to become pregnant during the course of the study.

- Use of any of the following drugs/supplements within the last 2 months:
> systemic antibiotics, antifungals, antivirals or antiparasitics (intravenous, intramuscular, or oral);
> corticosteroids (intravenous, intramuscular, oral, nasal or inhaled);
> cytokines;
> methotrexate or immunosuppressive cytotoxic agents;

- Chronic, clinically significant, or unstable (unresolved, requiring on-going changes to medical management or medication) pulmonary, cardiovascular, gastrointestinal, hepatic or renal functional abnormality, as determined by medical history, Type 1 diabetes, dialysis

- History of active cancer in the past 3 years except for squamous or basal cell carcinomas of the skin that have been medically managed by local excision

- Unstable dietary history as defined by major changes in diet during the previous month, where the subject has eliminated or significantly increased a major food group in the diet.

- Recent history of chronic excessive alcohol consumption defined as more than five 1.5-ounce servings of 80 proof distilled spirits, five 12-ounce servings of beer or five 5-ounce servings of wine per day; or > 14 drinks/week.

- Any confirmed or suspected condition/state of immunosuppression or immunodeficiency (primary or acquired) including HIV infection, multiple sclerosis and Graves' disease.

- Surgery of the GI tract, with the exception of cholecystectomy and appendectomy, in the past five years.

Any major bowel resection at any time.

- Regular/frequent use of smoking or chewing tobacco, e-cigarettes, cigars or other nicotine-containing products

- Regular use of prescription opiate pain medication

i) Describe your screening procedures, including how qualifying laboratory values will be obtained. If you are collecting personal health information prior to enrollment (e.g., telephone screening), please request a waiver of authorization for recruitment (in section 15).

The initial screening will take place using an on-line screening questionnaire in order to address the self-reported items of the inclusion/exclusion criteria.

j) Describe how you will be cognizant of other protocols in which participants might be enrolled. Please explain if participants will be enrolled in more than one study.

This question will be posed directly to participants during the on-line screening questionnaire and will also be a check-off item on the first page of the main study consent form that must be completed prior to enrollment into the study.

k) Payment/reimbursement. Explain the amount and schedule of payment or reimbursement, if any, that will be paid for participation in the study. Substantiate that proposed payments are reasonable and commensurate with the expected contributions of participants and that they do not constitute undue pressure on participants to volunteer for the research study. Include provisions for prorating payment. See payment considerations

No payments will be made to participants for participating in this study. Participants will receive on average 14 servings of meat or plant-based meat alternatives each week free-of-charge for four weeks.

l) Costs. Please explain any costs that will be charged to the participant.

Participants will be sent text message questionnaires daily. Standard text messaging rates will apply. No other costs will be charged to participants for participating in this study.

m) Estimate the probable duration of the entire study. Also estimate the total time per participant for: (i) screening of participant; (ii) active participation in study; (iii) analysis of participant data.

ENTIRE STUDY DURATION: We estimate that it will take about 12 months for 48 participants to complete the entire protocol and for us to complete the final analyses.

(i) Screening time:
2 weeks for each participant and includes time to complete online screening questionnaire (20 minutes), time to follow up (2-3 business days), orientation meeting (60 minutes), and teaching session (60 minutes)

(ii) Active participant time:
3 months for each participant and includes time for screening, intervention (8 weeks). While on study, participants will be spending, on average, 5-15 minutes each week on filling out questionnaires.

(iii) Data analysis time:
Approximately 4 additional months and will include time to send samples out for analyses or perform analyses in-house, as well as conduct final data quality checks and statistical analyses.

9. Risks

a) For the following categories include a scientific estimate of the frequency, severity, and reversibility of potential risks. Wherever possible, include statistical incidence of complications and the mortality rate of proposed procedures. Where there has been insufficient time to accumulate significant data on risk, a statement to this

effect should be included. (In describing these risks in the consent form to the participant it is helpful to use comparisons which are meaningful to persons unfamiliar with medical terminology.)

The risks of the Investigational devices.

Not applicable.

The risks of the Investigational drugs. Information about risks can often be found in the Investigator's brochure.

Not applicable.

The risks of the Commercially available drugs, reagents or chemicals. Information about risks can often be found in the package insert.

Not applicable.

The risks of the Procedures to be performed. Include all investigational, non-investigational and non-invasive procedures (e.g., surgery, blood draws, treadmill tests).

BLOOD DRAWS: There is a slight risk of infection, pain, or bruising at the blood site from blood sampling. This risk is minimized by having all blood sampling done by trained nurses at the Clinical Translational Research Unit of Stanford Hospital. The Tasso micro-sampling devices are minimally invasive devices that prick the arm and are essentially painless.

The risks of the Radioisotopes/radiation-producing machines (e.g., X-rays, CT scans, fluoroscopy) and associated risks.

Not applicable.

The risks of the Physical well-being.

No evidence of risk to physical well-being.

The risks of the Psychological well-being.

Some participants may feel discomfort answering certain questions on the study surveys.

The risks of the Economic well-being.

No evidence of risk to economic well-being.

The risks of the Social well-being.

No evidence of risk to social well-being.

b) If you are conducting international research, describe the qualifications/preparations that enable you to both estimate and minimize risks to participants. Provide an explanation as to why the research must be completed at this location and complete the [LINKFORINTERNATIONALREASEARCHFORM] International Research Form. If not applicable, enter N/A.

Not applicable.

c) Describe the planned procedures for protecting against and minimizing all potential risks. Include the means for monitoring to detect hazards to the participant (and/or to a potential fetus if applicable). Include steps to minimize risks to the confidentiality of identifiable information.

BLOOD DRAWS: All blood draws will be performed by the trained and experienced nurses at the CTRU at the Stanford Hospital.

IDENTIFIABLE INFORMATION: Each participant will be assigned a unique ID number upon first contact with our research group. All hard copy consent and case report forms will be filed in a locked cabinet, only the participant ID number will be listed on the file folder. Most electronic data will be captured either directly from participants or entered directly by study staff into our REDCap database. All other electronic data files will be kept on password protected and encrypted computers that comply with Stanford School of Medical HIPAA regulations. Consistent with Stanford University policy, all research staff will be required to complete the Human Subjects and HIPAA training modules for certification. Only research staff will have access to the hard copy files and electronic database and files. These methods have

proven successful at protecting confidentiality in our prior research. All data sent to team members for analysis will be coded.

d) **Explain the point at which the experiment will terminate. If appropriate, include the standards for the termination of the participation of the individual participant Also discuss plans for ensuring necessary medical or professional intervention in the event of adverse effects to the participants.**

We will terminate the study when the post-intervention data collection and data analysis have been completed. Based on our years of experience with similar research, we do not anticipate any need to terminate this study early.

10. Benefits

a) **Describe the potential benefit(s) to be gained by the participants or by the acquisition of important knowledge which may benefit future participants, etc.**

We cannot guarantee any benefits from participation in this study. The public may benefit from the potential scientific and public health impact of the knowledge that may result from this study. The potential benefits to the subject and to others outweigh the potential risks.

11. Privacy and Confidentiality

Privacy Protections

a) **Describe the setting and method (e.g. crowded waiting room, patient exam room, telephone or email communication) in which interactions will occur and how the privacy interests of participants will be maintained. Note, high risk data such as PHI must be sent via "Secure:" email per https://uit.stanford.edu/security/hipaa/email-policy Stanford policy.**

COMMUNICATION: Most communication with participants is done via email. Phone calls from staff are made from a private office.

CLINIC VISITS: For clinic visits, participants are seen in a private exam room and every effort is made to provide privacy with all procedures.

GROUP MEETINGS: The nature of our Orientation Meetings and Health Education classes is that they are group sessions and therefore cannot be "private." That said, only those individuals attending a specific session will know about the individuals attending that same session.

DATA COLLECTION: All on-line questionnaires can be filled out in the privacy of the individual's home. The dietary recall data are collected either over the telephone or online.

Confidentiality Protections

b) **Specify PHI (Protected Health Information). PHI is health information linked to HIPAA identifiers (see above). List BOTH health information AND HIPAA identifiers. If you are using STARR, use the Data Privacy Attestation to ensure that your request will match your IRB-approved protocol. Be consistent with information entered in section 15a.**

We will collect the following HIPAA identifiers as part of this research:
-Names

-Telephone numbers
-Address
-Email address
-Date of birth
-Medical Record Numbers

Additional health and personal data to be collected include the following:
-Basic demographics (i.e.: marital status, race, ethnicity, education)
-Self-reported disease status
-Clinical health information to include: weight, height, waist circumference, blood
pressure, various metabolic indices (i.e.: lipids, glucose, insulin)
-Other: dietary intake, physical activity, cognitive function, responses to psycho-social
questionnaires

As soon as the last participant completes the study, we will work with MedIRT to extract participant data from EPIC collected during their study related visits to the CTRU. We extract the following data: height, weight, waist circumference, blood pressure, and temperature.

After the close of a study, we are sometimes asked by outside investigators for coded data or samples for secondary/exploratory analyses. We do this only after having appropriate DUA's and MTA's in place. Specifically, we have been asked to share the following demographics: age, gender, race/ethnicity. We have also been asked to share the following health information: height, weight, blood pressure, waist circumference, and some blood results like lipid panels, etc.

c) **You are required to comply with University Policy that states that ALL electronic devices: computers (laptops and desktops; OFFICE or HOME); smart phones; tablets; external hard disks, USB drives, etc. that may hold identifiable participant data will be password protected, backed up, and encrypted. See http://med.stanford.edu/datasecurity/ for more information on the Data Security Policy and links to encrypt your devices.**

Stanford University IT approved platforms (https://uit.stanford.edu/guide/riskclassifications https://uit.stanford.edu/guide/riskclassifications) should be used for data management. Consult with your Department IT representative for more information.For data security policies and links to encrypt your devices see http://med.stanford.edu/irt/security and target=_blankhttp://www.stanford.edu/group/security/securecomputing/mobile_devices.html. Additionally, any PHI data on paper must be secured in a locked environment.

By checking this box, You affirm the aforementioned. Y

All hard paper copies of study data will be kept in a locked filing cabinet. File folders will only be labeled with the participant ID number.

All electronic data will be stored on password-secured, encrypted, AMIE-compliant, and Stanford-authorized computers. Specific data sets will be stored in REDCap, in Stanford Medicine Box, or on a Stanford-secured department server. Program manager will ensure that all staff have appropriate computers prior to starting work on any study.

Title : The Twins Nutrition Study (TwiNS): Vegan vs. Omnivore
Approval Period: 06/14/2022 - 12/31/2999

REDCap will serve as our primary Electronic Data Capture database. Most data will be captured directly from participants into REDCap as they complete REDCap-based questionnaires. Any additional data points (ie: blood values from external labs) for this study will be migrated into the main study REDCap project database upon receipt of data.

Diet Log Data: Cronometer is a food logging application, available for iOS, Android, or as a web-based platform, which tracks 60+ nutrients and boasts a database of 200,000+ foods. We will create accounts for the participants to minimize data going to Cronometer. Upon account activation, users may then log their diet, and/or exercise and biometrics. A Professional upgrade allows for institutions to access a study portal for participant management. Cronometer has on-board data protection officers who ensure the software abides by HIPAA compliance, GDPR, and basic principles to data security: encryption, least privileges, and industry best practices. All traffic from mobile app/browsers is encrypted, as are internal data and web servers, admin access and data sharing (for opt-in research portals). Passwords are hashed and salted.

Cognitive Function Data: Cambridge Cognition CANTAB tests are considered the gold standard in research, which cover the key domains of attention, memory, executive function, emotion and social cognition, and psychomotor speed. Software is designed for digital devices and provides automated data capture and scoring, and has translational utility. Data are kept secure by HTTPS 128-bit encryption for all data transfers, encrypted on tablets with 256-bit key file encryption, and stored remotely on a secure private cloud provisioned from HIPAA accredited Armor.

d) **Describe how data or specimens will be labeled (e.g. name, medical record number, study number, linked coding system) or de-identified. If you are de-identifying data or specimens, who will be responsible for the de-identification? If x-rays or other digital images are used, explain how and by whom the images will be de-identified.**

During data collection, it will be important for the study team to have access to the name, medical record number, and/or study ID so as to collect data from the correct participant. However, at the time of analysis all data will be coded and ONLY labeled with participant ID. Since all data will be housed in REDCap, the program manager will set up access and rights such that only coded data will be available for download and/or only provide coded CSV files.

All blood samples processed by the Stanford CTRU Lab are barcoded and labeled only with the study protocol number, participant ID number and appointment data. There is no PHI on the labels.

e) **Indicate who will have access to the data or specimens (e.g., research team, sponsors, consultants) and describe levels of access control (e.g., restricted access for certain persons or groups, access to linked data or specimens).**

The program manager, study coordinator, research assistants and interventionists will have full access to identifiable data so that data collection and study intervention can take place. The program manager will work with the statistician to provide data to the PI,

postdocs and other researchers that cannot be linked to specific individuals and will only contain a participant study ID number. All data will be housed in our REDCap database project that has the capability to export only coded data.

Participants will go to the CTRU for clinical visits, so the nursing staff will have full access to identifiable information. Samples will be processed by the CTRU/HIMC lab that has strict protocol to keep identifiable data safe and code data as early on in the process as possible.

Cronometer will store all diet log data. CANTAB will store all cognitive function data.

f) If data or specimens will be coded, describe the method in which they will be coded so that study participants' identities cannot be readily ascertained from the code.

All participants will be assigned a unique study ID upon entry into the study. These ID's will be assigned numerically based on time of consent form signing. If we enroll participants in multiple cohorts, we will use a different numbering scheme for each cohort (ie: cohort 1 = 1000's, cohort 2 = 2000's, etc)

On the samples provided by the CTRU/HIMC lab, only a bar code and study ID numbers will be used to identify samples.

g) If data or specimens will be coded, indicate who will maintain the key to the code and describe how it will be protected against unauthorized access.

Only the individuals on the research team that need to interact directly with participants will have access to file that links an individual with their study ID. This will all be stored in our REDCap database project. Anyone accessing that data for analysis purposes will only have access to coded data.

h) If you will be sharing data with others, describe how data will be transferred (e.g., courier, mail) or transmitted (e.g., file transfer software, file sharing, email). If transmitted via electronic networks, describe how you will secure the data while in transit.See http://www.stanford.edu/group/security/securecomputing/. http://www.stanford.edu/group/security/securecomputing/. Additionally, if you will be using or sharing PHI see https://uit.stanford.edu/security/hipaa https://uit.stanford.edu/security/hipaa.

Under no circumstances will be sharing PHI with other researchers. Our internal team will have access to coded data directly from the REDCap database project.

All research partners external to our immediate team will be only given access to the specific data files they request free of PHI. These files will be put into a secured Box account for them to access.

At the end of the study, we do send personalized results to participants so they know how their personal health changed throughout the course of the study.

i) How will you educate research staff to ensure they take appropriate measures to protect the privacy of participants and the confidentiality of data or specimens collected (e.g. conscious of oral and written communications, conducting insurance billing, and maintaining paper and electronic data)?

All staff are required to take the HIPAA training and remain in

compliance throughout the duration of the study. All staff will be required to provide documentation of completing this training prior to working with study participants or data. Research staff will be repeatedly reminded of the importance of protecting participant privacy and the confidentiality of data by the investigators in study staff meetings.

12. Potential Conflict of Interest

Investigators are required to disclose any outside interests that reasonably appear to be related/li to this protocol.

Outside Interest Tasks

Investigators	Role	Potential COI?	Date Outside Interest Answered	Date OPACS Disclosure Submitted	COI Review Determination
Christopher D Gardner	PD	N	12/05/2021		N/A
Justin L. Sonnenburg	COPD	N	12/16/2021		N/A
David Joel Maron	OP	N	03/15/2022		N/A

13. Consent Background

13. 1 Consent — Twins Online Screener Consent

Sponsor's Consent Version Number: (if any) :

a) Describe the informed consent process. Include the following.
- **i) Who is obtaining consent? (The person obtaining consent must be knowledgeable about the study.)**
- **ii) When and where will consent be obtained?**
- **iii) How much time will be devoted to consent discussion?**
- **iv) Will these periods provide sufficient opportunity for the participant to consider whether or not to participate and sign the written consent?**
- **v) What steps are you taking to minimize the possibility of coercion and undue influence?**
- **vi) If consent relates to children and if you have a reason for only one parent signing, provide that rationale for IRB consideration.**

i) Nobody will directly be obtaining consent as this is an initial online screening questionnaire. ii) Online when a potential participant begins entering data. iii) less than 20 min iv) yes v) There is no interaction with the potential participant at this point, so they can stop filling out the questionnaire at any time. vi) NA

b) What is the Procedure to assess understanding of the information contained in the consent? How will the information be provided to participants if they do not understand English or if they have a hearing impairment? See HRPP Chapter12.2 for guidance.

At the end of the questionnaire we ask if they filled it out or if someone did it for them. In addition, after completing the online screening questionnaire, all potential participants will receive a follow-up phone call to confirm understanding. For the purposes of this current study, we will only be enrolling participants that speak, read and understand English. This will be done because it is a small study and we will need to keep the health education classes small and in one language.

c) What steps are you taking to determine that potential participants have the capacity to participate in the decision-making process? If your study may enroll adults who are unable to consent, describe (i) how you will assess the capacity to consent, (ii) what provisions will be taken if the participant regains the capacity to consent,(iii) who will be used as a legally authorized representative, and (iv) what provisions will be made for the assent of the participant.

All potential participants will receive a phone call after completing the questionnaire which will allow the study team to assess overall competence with the study details.

13. 2 Consent Twins Main Study Consent

Sponsor's Consent Version Number: (if any) :

a) Describe the informed consent process. Include the following.

- **i) Who is obtaining consent? (The person obtaining consent must be knowledgeable about the study.)**
- **ii) When and where will consent be obtained?**
- **iii) How much time will be devoted to consent discussion?**
- **iv) Will these periods provide sufficient opportunity for the participant to consider whether or not to participate and sign the written consent?**
- **v) What steps are you taking to minimize the possibility of coercion and undue influence?**
- **vi) If consent relates to children and if you have a reason for only one parent signing, provide that rationale for IRB consideration.**

i) The PI or Study Coordinator will be obtaining consent and have participated in the Informed Consent Form Training. ii) Given the pandemic, this will likely be done over zoom in the evening during the week - Orientation Meeting (OM). During the OM, the PD will go over the study protocol and consent form in detail. All participants will be given an opportunity to ask questions during or after the presentation. During the presentation, a study team member will email the ppts a copy of the informed consent through RedCap. Participants will be able to follow along on the consent form and then sign if they feel comfortable doing so. The electronic version of the consent form on RedCap will mirror the consent approved by IRB. We will have a system in place to keep track of versions signed. iii) We will have over 60 min devoted to the consent discussion. iv) Yes, our experience has been that this is more than enough time. v) Everyone will be offered the opportunity to sign the consent form right away or leave the meeting to think about it. vi) NA

b) What is the Procedure to assess understanding of the information contained in the consent? How will the information be provided to participants if they do not understand English or if they have a hearing impairment? See HRPP Chapter12.2 for guidance.

Prior to getting to the Orientation Meeting where this main study consent will be signed, the study team will already have had several touch points with the participant - at a minimum via email and over the phone. This will allow amble opportunity for the team to assess a participant's understanding. In addition, the study team will make themselves available to answer any questions. For the purposes of this current study, we will only be enrolling participants that speak, read and understand English. This will be done because it is a small study and we will need to keep the health education classes small and in one language.

c) What steps are you taking to determine that potential participants have the capacity to participate in the decision-making process? If your study may enroll adults who are unable to consent, describe (i) how you will assess the capacity to consent, (ii) what provisions will be taken if the participant regains the capacity to consent,(iii) who will be used as a legally authorized representative, and (iv) what provisions will be made for the assent of the participant.

Again, prior to getting to the Orientation Meeting where this main study consent will be signed, the study team will already have had several touch points with the participant - at a minimum via email and over the phone. This will allow ample opportunities to assess competence.

14. Assent Background (less than 18 years of age)

15. HIPAA Background

15. 1 Waiver of Authorization for online screening questionniare Recruitment

a) **Describe the protected health information (PHI) needed to conduct screening or recruitment. PHI is health information linked to HIPAA identifiers. List BOTH health information AND HIPAA identifiers. If you are using STARR, use the Data Privacy Attestation to ensure that your request will match your IRB-approved protocol.**

As part of the online screening questionnaire, we need to collect some PHI from the ppt. This will include the following: name, email, address, phone number, date of birth, and some general information about their health so we can assess initial eligibility.

b) Please Answer:

Y **Do you certify that the use or disclosure of protected health information involves no more than a minimal risk to the privacy of individuals?**

Y **Do you certify that the research could not practically be conducted with out the waiver?**

Y **Do you certify that you have adequate written assurances that the protected health information will not be reused or disclosed to any other person or entity, except as required by law, for authorized oversight of the research project, or for other research for which the use or disclosure of protected health information would be permitted?**

Y **Do you certify that the research could not practically be conducted with out access to and use of the protected health information?**

c) **Please describe an adequate plan to protect any identifiers from improper use and disclosure.**

All online screening questionnaire data are collected directly into REDCap. Only the immediate study team has access to these data.

d) **Please describe an adequate plan to destroy the identifiers at the earliest opportunity consistent with conduct of the research, unless there is a health or research justification for retaining the identifiers or such retention is otherwise required by law.**

As this point in time, all the data collected are valuable to the research. Data collected allow us to follow up with ppts and complete additional analyses as opportunities present themselves. However, once data are exported from REDCap (without PHI), the REDCap project will be locked down and no longer accessible.

16. Attachments

Attachment Name	Attached Date	Attached By	Submitted Date
PerceivedStressScalePSS10	12/16/2021	taylerkl	
WHOFiveWellBeingIndex	12/16/2021	taylerkl	
IPAQ Physical Activity	12/16/2021	taylerkl	
PROMISSFV11GlobalHealth	12/16/2021	taylerkl	
GastrointestinalSymptoms	12/16/2021	taylerkl	
PROMISSFV10Fatigue	12/16/2021	taylerkl	

Title : The Twins Nutrition Study (TwiNS): Vegan vs. Omnivore
Approval Period: 06/14/2022 - 12/31/2999

ScreenerHealthHistory	12/16/2021	taylerkl	
ScreenerMedSupplList	12/16/2021	taylerkl	
Twins Flyer - Documentary	02/07/2022	taylerkl	
Twins Flyer - Study only	02/07/2022	taylerkl	
Protocol-63995_SSR	02/09/2022	ahorwege	
Barriers to Adherence	03/15/2022	taylerkl	
Cooking Behaviors and Habits	03/15/2022	taylerkl	
Factors to Improve Dietary Adherence	03/15/2022	taylerkl	
Preference to Diet Phase	03/15/2022	taylerkl	
Quality of Delivery Meals	03/15/2022	taylerkl	
Self Efficacy to Prepare and Cook	03/15/2022	taylerkl	
Self Rated Diet Adherence	03/15/2022	taylerkl	
Screener Participant Information	03/15/2022	taylerkl	
Screener Contact Information	03/15/2022	taylerkl	
Screener Diet and Lifestyle	03/15/2022	taylerkl	
Screener Study Requirements	03/15/2022	taylerkl	
Twin Data Collection Diagram	03/15/2022	taylerkl	
Twins Flyer_2022-03-17	03/17/2022	jlmorris	
Twins Flyer-Docu_2022-03-17	03/17/2022	jlmorris	
TwiNS_CANTAB_section16	03/21/2022	jlmorris	
TwiNS Data Collection Diagram 2022-03-30	03/30/2022	jlmorris	
Twins Flyer_2022-04-03	04/03/2022	jlmorris	
Twins Flyer-Docu_2022-04-03	04/03/2022	jlmorris	
DietarySatisfaction	06/02/2022	taylerkl	
TwiNS Week 8 Questionnaires	06/02/2022	taylerkl	
TwiNS End Questionnaires	06/02/2022	taylerkl	

Title : The Twins Nutrition Study (TwiNS): Vegan vs. Omnivore
Approval Period: 06/14/2022 - 12/31/2999

Obligations

The Protocol Director agrees to:

- Adhere to principles of sound scientific research designed to yield valid results
- Conduct the study according to the protocol approved by the IRB
- Be appropriately qualified to conduct the research and be trained in Human Research protection, ethical principles, regulations, policies and procedures
- Ensure all Stanford research personnel are adequately trained and supervised
- Ensure that the rights and welfare of participants are protected including privacy and confidentiality of data
- Ensure that, when de-identified materials are obtained for research purposes, no attempt will be made to re-identify them.
- Disclose to the appropriate entities any potential conflict of interest
- Report promptly any new information, modification, or unanticipated problems that raise risks to participants or others
- Apply relevant professional standards.

Any change in the research protocol must be submitted to the IRB for review prior to the implementation of such change. Any complications in participants or evidence of increase in the original estimate of risk should be reported at once to the IRB before continuing with the project. Inasmuch as the Institutional Review Board (IRB) includes faculty, staff, legal counsel, public members, and students, protocols should be written in language that can be understood by all Panel members. The investigators must inform the participants of any significant new knowledge obtained during the course of the research.

IRB approval of any project is for a maximum period of one year. For continuing projects and activities, it is the responsibility of the investigator(s) to resubmit the project to the IRB for review and re-approval prior to the end of the approval period. A Notice to Renew Protocol is sent to the Protocol Director 7 weeks prior to the expiration date of the protocol.

https://stanfordmedicine.box.com/shared/static/qbsi8u8h47qsotxhdpuzz50xlrqa0sgo.pdf Report promptly any new information, complaints, possibly serious and/or continuing noncompliance, or unanticipated problems involving risks to participants or others.

All data including signed consent form documents must be retained for a minimum of three years past the completion of the research. Additional requirements may be imposed by your funding agency, your department, or other entities. (Policy on Retention of and Access to Research Data, Research Policy Handbook, http://doresearch.stanford.edu/policies/research-policy-handbook/conduct-research/retention-and-access-research-data)

APPROVAL LETTER/NOTICE NOTE: List all items (verbatim) that you want to be included in your approval letter (e.g., Amendment date, Investigator's Brochure version, consent form(s) version(s), advertisement name, etc.) in the box below.

Y By checking this box, I verify that I, as the Protocol Director (PD) responsible for this research protocol, have read and agree to abide by the above obligations, or that I have been delegated authority by the PD to certify that the PD has read and agrees to abide by the above obligations.

Statistical Analysis Plan (SAP)

Title: Twins Nutrition Study (TwiNS): Vegan vs. Omnivore

CRU/Department/Division/Center: Stanford Prevention Research Center

IRB Number: 63995

Clinical Trials: NCT05297825

Primary Investigator: Christopher Gardner

Collaborative Lead: Matthew Landry

Co-authors (if know):

Analysis Biostatistician(s): Matthew Landry, Cate Ward

Biostatistics Supervisor: Kristen Cunanan

Lead Biostatistician: Matthew Landry

Subject Matter Expert: Christopher Gardner

Original Creation Date:

Version Date: October 17, 2022

Project Folder Location: StanfordMed Box

Project Goal(s): Manuscript

Submission Deadline(s): TBD

Investigator Agreement

- ☐ All statistical analyses included in an abstract or manuscript should reflect the work of the biostatistician(s) listed on this SAP. No changes or additional analyses should be made to the results or findings without discussing with the project biostatistician(s).
- ☐ All biostatisticians on this SAP should be given sufficient time to review the full presentation, abstract, manuscript, or grant and be included as co-authors on any abstract or manuscript resulting from the analyses.
- ☐ If substantial additional analysis is necessary or the aims of the project change, a new SAP will need to be developed.
- ☐ If you engaged the CTSA's BERD please ensure you cite UL1TR003142 award when disseminating work. If your study was cancer-related, please ensure you cite P30CA124435 award when disseminating work. If your study was diabetes-related, please ensure you cite P30DK116074 award when disseminating work. These publications should also be submitted to PubMed Central.

☐ I have reviewed the SAP and understand that any changes must be documented.

Acknowledged by:

Lead Principal Investigator: Lead Biostatistician:

________________________ ________________________

Date: ___________________ Date: __________________

____

Activity Log:

1. Study Overview

Background/Introduction: A vegan diet has been promoted as having a much lower environmental impact, but there is some controversy about the health effects of following a vegan diet. Some believe that this plant-based diet can help prevent many of the chronic diseases that affect us, such as heart disease, cancer, and Alzheimer's disease and would also help in managing body weight. Others claim that the vegan diet does not provide all the required nutrients, such as adequate amounts of protein, vitamin B12 and the minerals iron and calcium, and thus it would not promote optimal health. This study is designed to investigate the health impact of a vegan diet compared to an omnivorous diet. We plan to study these diets in twins, where one twin follows a vegan diet and the other twin follows an omnivorous diet, thus we control for genetic differences that might impact the effect of the diet.

Twenty-two pairs of identical twins will be randomized so that one twin will followed the vegan diet, and the other twin will follow the omnivorous diet for 8 weeks. Participants will receive pre-made meals delivered to their home for the first 4 weeks. They will be responsible for their own meals the last 4 weeks. Participants will have 3 in-person clinic visits (baseline, 4 weeks, and 8 weeks), as well as provide stool and micro-samples of blood from home. Changes in food patterns and behaviors and dietary intake assessment will be addressed using multiple tools employed successfully over dozens of studies by the Stanford Nutrition Studies Group.

1.1 Study Aims

1. To investigate a difference in the change in lipids (and other cardiometabolic risk factors) between a vegan and omnivorous diet after 8 weeks among identical twins
    a. If significant, to investigate a difference in the change in lipids (and other clinical risk factors) between a vegan and omnivorous diet after 4 weeks among identical twins
2. To investigate a difference in the change in inflammatory markers between a vegan and omnivorous diet after 8 weeks among identical twins
    a. If significant, to investigate a difference in the change in lipids (and other clinical risk factors) between a vegan and omnivorous diet after 4 weeks among identical twins
3. To investigate a difference in the change in alpha diversity between a vegan and omnivorous diet after 8 weeks among identical twins
    a. If significant, to investigate a difference in the change in lipids (and other clinical risk factors) between a vegan and omnivorous diet after 4 weeks among identical twins

1.2 Study Hypotheses

1.2.1 Primary Hypotheses

- The change in lipids will be different for participants on vegan diet compared to those on omnivorous diet
    - A difference can be seen as early as 4 weeks

1.2.2 Secondary Hypotheses

- The change in inflammatory markers will be different for participants on vegan diet compared to those on omnivorous diet
    - A difference can be seen as early as 4 weeks
- The change in alpha diversity will be different for participants on vegan diet compared to those on omnivorous diet
    - A difference can be seen as early as 4 weeks

2. Study Population

2.1 Inclusion Criteria

- Age 18+
- 1/2 of a pair of twins that will both be participating

- Willing to consume a plant-based diet (vegetables, fruit, whole grains, legumes, etc.)
- Willing to consume meat/eggs (beef, pork/sausage, chicken, eggs) >= 1 time a day
- Willing to consume dairy (milk, yogurt, cheese) >= 1 time a day

2.2 Exclusion Criteria

- Weight < 110 lb
- BMI >= 40
- LDL-C > 190 mg/dL
- Systolic Blood Pressure > 160 mmHg OR Diastolic blood pressure > 90 mmHg
- Pregnant, lactating or planning to become pregnant during the course of the study.
- Use of any of the following drugs/supplements within the last 2 months:
  - systemic antibiotics, antifungals, antivirals or antiparasitics (intravenous, intramuscular, or oral);
  - corticosteroids (intravenous, intramuscular, oral, nasal or inhaled);
  - cytokines;
  - methotrexate or immunosuppressive cytotoxic agents.
- Chronic, clinically significant, or unstable (unresolved, requiring on-going changes to medical management or medication) pulmonary, cardiovascular, gastrointestinal, hepatic or renal functional abnormality, as determined by medical history, Type 1 diabetes, dialysis.
- History of active cancer in the past 3 years except for squamous or basal cell carcinomas of the skin that have been medically managed by local excision
- Unstable dietary history as defined by major changes in diet during the previous month, where the subject has eliminated or significantly increased a major food group in the diet.
- Recent history of chronic excessive alcohol consumption defined as more than five 1.5-ounce servings of 80 proof distilled spirits, five 12-ounce servings of beer or five 5-ounce servings of wine per day; or > 14 drinks/week.
- Any confirmed or suspected condition/state of immunosuppression or immunodeficiency (primary or acquired) including HIV infection, multiple sclerosis and Graves' disease.
- Surgery of the GI tract, with the exception of cholecystectomy and appendectomy, in the past five years. Any major bowel resection at any time.
- Regular/frequent use of smoking or chewing tobacco, e-cigarettes, cigars or other nicotine-containing products
- Regular use of prescription opiate pain medication

2.3 Data Acquisition

Study design: Randomized controlled trial with parallel assignment
Contact information for team member responsible for data collection / acquisition: Tayler Hennings
Where dataset is stored: StanfordMed Box

3. Outcomes, Exposures, and Additional Variables for Interest

3.1 Primary Outcome(s)

Outcome	Description	Variables and source	Specifications
LDL	Measured at baseline, 4 and 8 weeks		Continuous, measured in mg/dL

3.2 Secondary Outcome(s)

Outcome	Description	Variables and source	Specifications
HDL	Measured at baseline, 4 and 8 weeks		Continuous, measured in mg/dL
Triglycerides	Measured at baseline, 4 and 8 weeks		Continuous, measured in mg/dL
Fasting Insulin	Measured at baseline, 4 and 8 weeks		Continuous, measured in μIU/mL
Fasting Glucose	Measured at baseline, 4 and 8 weeks		Continuous, measured in mg/dL
TMAO	Measured at baseline, 4 and 8 weeks		Continuous, measured in μ/M
Weight	Measured at baseline, 4 and 8 weeks		Continuous, measured in kg

Serum B-12	Measured at baseline, 4 and 8 weeks		Continuous, measured in pg/ml

3.3 Exploratory Outcome(s)

Outcome	Description	Variables and source	Specifications
Inflammation Markers	Measured at baseline, 4 and 8 weeks		Continuous
Alpha Diversity	Measured at baseline, 4 and 8 weeks		Continuous, number of observed sequence variants ("species") determined by standard 16S rRNA amplicon sequencing (V3-V5 region followed by DADA2 to define error-corrected sequence variants). Note: *Higher alpha diversity is better. The units are the # of sequence variants*

4. Statistical Analysis Plan

Data management and data quality check:
For all variables (demographic and clinical variables), we will present spaghetti plots by arm. Visually we will examine the plots for any possible data errors (e.g., implausible value, implausible change between time points, etc.) or extreme outliers. Additionally, for all variables, we will present histograms, by arm, as well as, overall participants. Here, we will examine the distributions for skewness or multi-modality; and determine if any variable transformations are necessary for modeling. Lastly, we will present the available sample size for each variable at each time point, within a study arm.

4.1 Demographic and Clinical Characteristics ("Table 1")

Demographic variables: age (continuous), sex, race/ethnicity, twins housing arrangement (dichotomous: living together or separate)
Clinical variables: LDL-C, HDL-C, triglycerides, fasting insulin, fasting glucose, TMAO, weight, serum B12
Process variables: caloric intake, macronutrient intake

Mean (standard deviation) or n (percent) will be presented for continuous and categorical variables, respectively. Variables with skewed distributions will also be presented as median (interquartile range) -- present on second line or in text or remove mn(sd). Table 1 will present summary statistics, by arm. We will present absolute standardized differences (ASDs) by study arm to investigate if there are any small, medium, or large differences between arms (0.2, 0.5, or 0.8, respectively). Furthermore, given participants are identical twin pairs, we will present summary statistics for baseline variable differences within a twin pair. We note process variables will be used to examine diet adherence; participants on a vegan diet should have no animal protein and cholesterol and higher fiber intake (than baseline).

4.2 Analysis Plan for Aim 1
For the primary analysis, we want to investigate a difference in the change from baseline for LDL between a vegan and carnivorous diet after 8 weeks, among identical twins. The primary outcome will be absolute LDL. The primary analysis will include all available data. A linear mixed model will be used and include fixed effects for diet and time (baseline as reference) and an interaction effect for diet by time, and a random effect for twin pair, to account for the correlation between identical twins (i.e. random intercept allows intercept to vary for each twin pair). A Wald test will be used to evaluate a significant difference in diet at 8 weeks from baseline (interaction term), at a 5% significance level. If a significant difference at 8 weeks is observed, we will use a Wald test to evaluate a significant difference in diet at 4 weeks from baseline. Here, we want to determine if the improvement in LDL can be observed as soon as 4 weeks on a vegan diet. Finally, we will present model estimates (95% CI) for diet at 4 and 8 weeks.

We will implement the following secondary analyses. For each secondary outcome, we will evaluate a model similar to the primary model as described above. In Table 1, if any demographic, clinical, or process variables display more than small differences between arms (as measured by ASD > 0.2), we will evaluate the primary model as described

above, while adjusting for the variable(s).

Lastly, we will consider the following exploratory analyses. For academic purposes, we will implement a conservative approach that ignores the correlation within twin pairs and perform a two-sample t-test for the primary and secondary outcomes. Additionally, we will implement an overly liberal approach that incorrectly assumes identical twins can represent the same experimental unit in a matched t-test.

We note 4 pairs of twins were selected for media representation on a Netflix show; subsequently, these participants were supervised more than other participants. In a sensitivity analysis, we will run the primary analyses, excluding these 4 pairs of twins.

5. Limitations

- We note 4 pairs of twins were selected for media representation on a Netflix show; subsequently, these participants were supervised more than other participants.
- Participants on a vegan diet may eat fewer calories

References

1. Gardner CD, Offringa LC, Hartle JC, et al. Weight loss on low-fat vs. low-carbohydrate diets by insulin resistance status among overweight adults and adults with obesity: A randomized pilot trial. *Obesity* 2016;24(1):79-86.
