# Supplementary material for: Cardiometabolic Effects of Omnivorous vs Vegan Diets in Identical Twins: A Randomized Clinical Trial
Source: JAMA Netw Open. 2023 Nov 30;6(11):e2344457. doi: 10.1001/jamanetworkopen.2023.44457 (PMC10690456; doi:10.1001/jamanetworkopen.2023.44457)
Supplement: Supplement 2. — eMethods. Supplementary Methods eResults. Supplementary Results eAppendix. Plant-Based Dietary Intervention Reporting Checklist eFigure 1. TwiNS Study Design eTable 1. Twin Lifestyle Behaviors, n = 42 (21 Pairs of Twins) eFigure 2. Macronutrient Distribution by Phase and Diet eTable 2. Macronutrient Distribution by Phase and Diet eFigure 3. Fat Distribution by Phase and Diet eTable 3. Fat Distribution by Phase and Diet eFigure 4. Grain Distribution by Phase and Diet eTable 4. Grain Distribution by Phase and Diet eFigure 5. Protein Distribution by Phase and Diet eTable 5. Protein Distribution by Phase and Diet eFigure 6. Carbohydrate Distribution by Phase and Diet eTable 6. Carbohydrate Distribution by Phase and Diet eFigure 7. Dietary Cholesterol Distribution by Phase and Diet eTable 7. Dietary Cholesterol Distribution by Phase and Diet eFigure 8. Dietary Vitamin B12 Distribution by Phase and Diet eTable 8. Dietary Vitamin B12 Distribution by Phase and Diet eFigure 9. Dietary Iron Distribution by Phase and Diet eTable 9. Dietary Iron Distribution by Phase and Diet eFigure 10. Vegetable Servings Distribution by Phase and Diet eTable 10. Vegetable Servings Distribution by Phase and Diet eFigure 11. Animal-Based Protein Distribution by Phase and Diet eTable 11. Animal-Based Protein Distribution by Phase and Diet eFigure 12. Meat Alternatives Distribution by Phase and Diet eTable 12. Meat Alternatives Distribution by Phase and Diet eTable 13. Diet Satisfaction (D-Sat28) of Participants by Diet Assignment and Phase eTable 14. Self-Efficacy to Plan, Shop, Cook, and Choose Meals by Diet Assignment and Phase eTable 15. Diet Preferences of Participants by Diet Assignment eTable 16. Perceptions of Delivered, Pre-Prepared Meals by Diet Assignment eTable 17. Barriers to Adherence to Study Eating Patterns by Diet Assignment eTable 18. Factors that Improve Dietary Adherence and Sustainability by Diet Assignment eTable 19. Average Self-Rated Dietary Adherence by Diet Assignment an [file jamanetwopen-e2344457-s002.pdf]

Supplementary Online Content

Landry MJ, Ward CP, Cunanan KM, et al. Cardiometabolic effects of omnivorous vs vegan diets in identical twins: a randomized clinical trial. *JAMA Netw Open*. 2023;6(11):e2344457. doi:10.1001/jamanetworkopen.2023.44457

eMethods. Supplementary Methods
eResults. Supplementary Results
eAppendix. Plant-Based Dietary Intervention Reporting Checklist
eFigure 1. TwiNS Study Design
eTable 1. Twin Lifestyle Behaviors, n=42 (21 Pairs of Twins)
eFigure 2. Macronutrient Distribution by Phase and Diet
eTable 2. Macronutrient Distribution by Phase and Diet
eFigure 3. Fat Distribution by Phase and Diet
eTable 3. Fat Distribution by Phase and Diet
eFigure 4. Grain Distribution by Phase and Diet
eTable 4. Grain Distribution by Phase and Diet
eFigure 5. Protein Distribution by Phase and Diet
eTable 5. Protein Distribution by Phase and Diet
eFigure 6. Carbohydrate Distribution by Phase and Diet
eTable 6. Carbohydrate Distribution by Phase and Diet
eFigure 7. Dietary Cholesterol Distribution by Phase and Diet
eTable 7. Dietary Cholesterol Distribution by Phase and Diet
eFigure 8. Dietary Vitamin B12 Distribution by Phase and Diet
eTable 8. Dietary Vitamin B12 Distribution by Phase and Diet
eFigure 9. Dietary Iron Distribution by Phase and Diet
eTable 9. Dietary Iron Distribution by Phase and Diet
eFigure 10. Vegetable Servings Distribution by Phase and Diet
eTable 10. Vegetable Servings Distribution by Phase and Diet
eFigure 11. Animal-Based Protein Distribution by Phase and Diet
eTable 11. Animal-Based Protein Distribution by Phase and Diet
eFigure 12. Meat Alternatives Distribution by Phase and Diet
eTable 12. Meat Alternatives Distribution by Phase and Diet
eTable 13. Diet Satisfaction (D-Sat28) of Participants by Diet Assignment and Phase
eTable 14. Self-Efficacy to Plan, Shop, Cook, and Choose Meals by Diet Assignment and Phase
eTable 15. Diet Preferences of Participants by Diet Assignment
eTable 16. Perceptions of Delivered, Pre-Prepared Meals by Diet Assignment
eTable 17. Barriers to Adherence to Study Eating Patterns by Diet Assignment
eTable 18. Factors that Improve Dietary Adherence and Sustainability by Diet Assignment
eTable 19. Average Self-Rated Dietary Adherence by Diet Assignment and Phase
eTable 20. Cardiovascular Health Outcomes at the End of 4-weeks and Main Effect Model Estimates, Standard Errors, and 95% Confidence Interval for Primary and Secondary Outcome Analysis
eFigure 13. Sensitivity Analysis of the Change in Trimethylamine N-Oxide (TMAO) With Three Outliers Removed, (Mean ± SE).
eTable 21. Paired T-Tests of Cardiovascular Health Outcomes at the End of 8-Weeks
eTable 22. Unpaired T-Tests of Cardiovascular Health Outcomes at the End of 8-Weeks
eTable 23. Average Macronutrient and Micronutrient Composition of Trifecta Food Delivery Meals by Meal Type and Diet Assignment
eReferences

This supplementary material has been provided by the authors to give readers additional information about their work.

/

© 2023 Landry MJ et al. *JAMA Network Open.*

eMethods. Supplementary Methods

Inclusion Criteria and Recruitment

Inclusion criteria included being ≥18 years of age, being an identical twin with both twins willing to participate, body mass index <40, and LDL-C <190 mg/dL. Participants also had to be willing and able to consume either a plant-based vegan diet (i.e., vegetables, fruit, whole grains, legumes), or meat/eggs (i.e., beef, pork/sausage, chicken, eggs) ≥1 time a day, and dairy (i.e., milk, yogurt, cheese) ≥1 time a day depending on their diet randomization. The major criteria for exclusion were having uncontrolled hypertension or metabolic disease; diabetes; cancer; heart, renal, or liver disease; and being pregnant or lactating. Individuals were excluded if taking hypoglycemic, lipid-lowering, antihypertensive, psychiatric, or other medications known to affect body weight or energy expenditure.

Initially, 135 participants completed an online screening questionnaire; 57 were excluded. Following a phone screen in which 30 individuals were excluded, 48 participants attended a study orientation and signed informed consent. Of the 48 consented participants, 4 dropped from the study following orientation and prior to being randomized. 44 participants (22 pairs of twins) were randomized (22 to the vegan group and 22 to the omnivorous group). One pair of twins dropped from the study after the week 4 timepoint. 42 participants (21 pairs of twins) completed the 8-week study.

Dietary Intervention

The intervention included three classes over the course of the two phases led by health educators via Zoom (Zoom Video Communications). The first class (Week 0) introduced the study eating

© 2023 Landry MJ et al. *JAMA Network Open.*

plan, healthful eating principles, and intake targets to complement three daily meals provided via meal delivery service. The second class (Week 4) reinforced healthful eating principles and provided guidance and tools for Phase 2, during which participants would cook meals on their own. The third class (Week 6) was midway through Phase 2 and discussed the successes and strategized around challenges of cooking on their own. High-quality, healthy, food intake was emphasized for both omnivorous and vegan eating plans during each session. Classes were complemented by email summaries as well as additional email check-ins during Weeks 2 and 7. Participants were able to contact health educators throughout the study for additional support.

For the omnivorous group, the health educators instructed the study participants to eat enough animal products daily in order to differentiate from the vegan group. Specifically, this included targets of 6-8 ounces of meat, fish, or poultry, 1 egg, and 1.5 servings of dairy each day, on average. Aside from animal products, targets included 3 servings of vegetables, 2 servings of fruit, and 6 servings of grains or starchy vegetables each day. For the vegan group, the health educators instructed the study participants to avoid all animal products for the course of the study. Specific targets included 6+ servings of vegetables, 3 servings of fruit, 5 servings of legumes, nuts, seeds, or vegan meat, and 6 servings of grains or starchy vegetables each day.

Many definitions of a plant-based diet exist; thus, it is of paramount importance to describe the dietary intervention in detail to facilitate comparison and reproducibility of studies[1]. To facilitate this, a plant-based dietary intervention reporting checklist[2] is provided in **Supplementary Materials**.

Participants living within the same household as their twin were not prohibited from discussing participation in the study with their twin; however, they were instructed to not share any food or meals with their twin. During the self-provided food phase, twins were reminded to independently prepare and cook separate meals from their twin to maintain adherence to their assigned diet.

24-hours Dietary Recalls

Dietary intake data gathered by interview was governed by a multiple pass interview approach. Five distinct passes provided multiple opportunities for the participant to recall food intake. A "Foods Amounts Booklet" was used to assist participants with portion size estimation. Recalls conducted took between 15–45 min to complete, depending upon the complexity of the intake. Diet assessors had a wide range of availability to accommodate participants' varying schedules, from early morning through evening. Generous staff time was required for capturing participant recalls. Quality assurance was performed on all dietary recall data by research staff trained in NDS-R.

Food Delivery

Food deliveries were modified as needed on an individual basis to allow for participants' self-reported food allergies and food aversions (nuts (n=6), milk (lactose) (n=2), anchovies (n=1), fish (n=1), mushrooms (n=1), avocado (n=1)).

© 2023 Landry MJ et al. *JAMA Network Open.*

Clinical Measures

Height was measured at the first visit to the nearest 0.1 cm using a Seca wall-mounted stadiometer. Body weight was recorded without shoes to the nearest 0.1 kg using a calibrated Scale-Tronix clinical scale. Fasting blood draws were completed at the CTRU via venipuncture by trained nurses or phlebotomists. Plasma glucose levels were measured within 2 hours of collection of fresh blood using a Nova Biomedical glucose analyzer. Plasma insulin and lipid concentrations were analyzed at the Core Laboratory for Clinical Studies (Washington University, St. Louis, MO) [3-6]. Insulin was analyzed by radioimmunoassay. Plasma total cholesterol and triglycerides were measured by enzymatic method and HDL cholesterol was measured by direct method on the Roche cobas c501 using Roche cobas reagents. LDL cholesterol was calculated using the Friedewald equation [7]. If triglycerides were >400 mg/dL, LDL cholesterol was measured using a direct method on the Roche cobas c501 using Sekisui reagents. Serum TMAO was measured by LC with tandem MS (Cleveland HeartLab, Cleveland, OH) [8].

Dietary Preferences, Satisfaction, and Barriers to Adherence

At baseline and at the end of each 4-week phase, we examined diet satisfaction using the D-SAT28[9]. At the end of the study, participants were asked to self-report their self-efficacy to plan, shop, cook, and choose meals, barriers to adherence each study phase, and factors to improve dietary adherence and sustainability, quality of delivered, pre-prepared meals, and diet preferences. Participants were also asked to self-rate their dietary adherence during the study [10].

© 2023 Landry MJ et al. *JAMA Network Open.*

Sensitivity Analyses

We considered several prespecified sensitivity analyses. First, primary and secondary outcomes which were significantly different at 8-weeks were also examined to determine if changes in the outcome could be observed as soon as 4 weeks. Four pairs of twins were selected to be featured in a documentary. Subsequently, these participants were supervised more than other participants and encouraged more than others to exercise, both of which may have influenced lifestyle behaviors and adherence to study protocols. In a second sensitivity analysis we conducted the primary analysis, excluding these 4 pairs of twins. Lastly, we considered the following exploratory analysis. For academic purposes, we implemented a conservative approach that ignores the correlation within twin pairs and performed a two-sample t-test for the primary and secondary outcomes. Additionally, we implemented an overly liberal approach that incorrectly assumes identical twins can represent the same experimental unit in a matched t-test.

TMAO sensitivity analysis

These values were reviewed for plausibility as well as participant diet records reflecting intake on the same day or day prior to the participant's blood draw. For two participants, dietary recalls reflecting the 24-hours prior to the blood draw reflected large intakes of TMAO-containing foods (e.g., fish) known to increase serum TMAO post consumption[11]. In a post-hoc sensitivity analysis, we removed these values from the analysis, leaving in the participants' other values at other timepoints.

© 2023 Landry MJ et al. *JAMA Network Open.*

eResults. Supplementary Results

Servings of vegetables increased in both diet arms during the food-delivery and self-provided phase of the intervention (**Supplemental Figure 10 and Supplemental Table 10**). As intended, servings of animal-based protein significantly decreased, to nearly none, among vegan participants during the food-delivery and self-provided phase of the intervention (**Supplemental Figure 11 and Supplemental Table 11**). Servings of animal-based protein increased among omnivore participants, with poultry, beef, and eggs being main contributors, during both the food-delivery and self-provided phase of the intervention. Servings of meat-alternatives increased among vegan participants during the food-delivery and self-provided phase of the intervention (**Supplemental Figure 12 and Supplemental Table 12**). Meat alternatives, including tofu, tempeh, soy nuts, and veggie burgers, were the largest source followed by nuts and seeds. Interestingly, intake of meat alternatives decreased during the food delivery and self-provided phase among omnivores compared to baseline levels.

Sensitivity Analysis – Featured Twins. In a prespecified sensitivity analyses, we conducted the primary analysis, excluding 4 pairs of twins that were selected for media representation in a documentary. We observed no changes to primary or secondary outcomes.

Diet Satisfaction – Within the vegan diet arm, diet satisfaction decreased on every scale apart from the healthy lifestyle scale, which increased at weeks 4 and 8 relative to baseline. Notably participants assigned to the vegan diet arm reported the largest decrease in diet satisfaction when eating out at weeks 4 and 8 relative to baseline. Among participants in the omnivorous diet arm,

© 2023 Landry MJ et al. *JAMA Network Open.*

diet satisfaction either increased at weeks 4 and 8 or was maintained from baseline reported levels.

Dietary Intake – On average, reported total energy intake decreased during the food delivery and self-provided phases within both diet arms (**Supplemental Figure 2 and Supplemental Table 2**). Both diet arms increased the percent of fat from unsaturated foods compared to saturated foods and the percentage of total calories from fat (**Supplemental Figure 3** and **Supplemental Table 3**). Intake of whole grains (in ounce equivalent servings) increased while intake of refined grains decreased within both groups, particularly during the food delivery phase of the intervention (**Supplemental Figure 4 and Supplemental Table 4**). Protein intake, as a percentage of calories, decreased among vegan participants and increased among omnivorous participants during the food delivery and self-provided phases of the intervention. Vegan participant's intake of protein overwhelmingly came from plant-based sources, while the majority of omnivore participant's intake of protein came from animal-based sources (**Supplemental Figure 5 and Supplemental Table 5**). Carbohydrate intake, in grams, was higher among vegan than omnivore participants. Grams of added sugar decreased from baseline levels among participants in both groups while grams of fiber (soluble and insoluble) increased among participants in both groups, particularly among vegan participants (**Supplemental Figure 6 and Supplemental Table 6**).

Intake of specific nutrients (dietary cholesterol, vitamin B12, and iron) by diet arm and per intervention phase are provided in **Supplemental Figures 7-9** and **Supplemental Tables 7-9**. Intake of dietary cholesterol significantly decreased among vegan participants, dropping from

245 mg to <16mg during the food-delivery and self-provided phase of the intervention (**Supplemental Figure is 7** and **Supplemental Table 7**). Not surprisingly, intake of dietary cholesterol was lower among vegan compared to omnivore participants during the food-delivery and self-provided phase of the intervention. Intake of vitamin B12 significantly dropped among vegan compared to omnivore participants during the food-delivery and self-provided phase of the intervention (**Supplemental Figure 8 and Supplemental Table 8**). Omnivore participants marginally increased intake of vitamin B12 during the food-delivery and self-provided phase of the intervention relative to baseline. Surprisingly, intake of iron significantly increased among vegan participants relative to baseline and intake was significantly higher for vegan compared to omnivore participants throughout the study, which we have observed before in previous studies possibly due to fortification of refined grains[12] (**Supplemental Figure 9 and Supplemental Table 9).** We note that iron intake was lower among vegan participants during the self-provided phase compared to the food-delivery phase.

Participants irrespective of diet arm self-reported having greater self-efficacy to plan, shop, cook, and choose meals that were reflective of their diet arm compared to baseline (**Supplemental Table 14**). One exception to this general trend were vegan participants at week 8 who reported lower self-efficacy to choose foods at restaurants that follow eating pattern recommendations compared to baseline.

Participants were split evenly when asked if they found it easier to adhere to dietary recommendation when receiving the delivered, pre-prepared meals or when preparing their own meals (**Supplemental Table 15**). Nearly all respondents in the vegan diet arm (91%) and the

© 2023 Landry MJ et al. *JAMA Network Open.*

majority of participants in the omnivorous arm (67%) reported that after the intervention they plan to continue to follow some but not all recommendations for their assignment diet.

Overall, participants had similar positive perceptions of delivered, pre-prepared meals that were provided during the first four weeks of the intervention, irrespective of diet arm (**Supplemental Table 16**). Participants were asked what barriers to adherence they experienced during the intervention, and responses were similar between diet arms (**Supplemental Table 17**). A "busy lifestyle" was the highest reported barrier among participants. Other noted barriers were time required to prepare meals and foods that spoil before getting used.

Based on prior dietary interventions conducted by the research team, several intervention components (meeting with a health educator, receiving delivered, pre-prepared meals, and having a digital method to log dietary intake) were offered to participants to improve dietary adherence during the intervention (**Supplemental Table 18**). A majority of participants (~69%) either strongly agree or agree that meeting regularly with the study's health educator while receiving delivered, pre-prepared meals during the first 4 weeks of the study helped them adhere to eating pattern recommendation in the subsequent 4 weeks. Additionally, about 57% of participants reported that receiving the delivered, pre-prepared meals helped them become more familiar with their assigned diet. Approximately 52% of participants reported that logging their dietary intake on Cronometer helped them adhere to eating pattern recommendations. Self-reported dietary adherence was higher among twins assigned to the vegan diet compared to omnivorous at weeks 4 and 8 (**Supplemental Table 19**).

© 2023 Landry MJ et al. *JAMA Network Open.*

eAppendix. Plant-Based Dietary Intervention Reporting Checklist

Many concepts of plant-based diet exist; thus, it is of paramount importance to describe the dietary intervention in detail to facilitate comparison and reproducibility of studies.

Checklist: Storz, M.A. What makes a plant-based diet? a review of current concepts and proposal for a standardized plant-based dietary intervention checklist. *Eur J Clin Nutr* 76, 789–800 (2022). https://doi.org/10.1038/s41430-021-01023-z

1. **The plant-based diet in the present study included:**
   ☐Meat ☐Poultry ☐Fish/Seafood ☐Dairy ☐Eggs ☒Honey
2. **If animal products were included, at what frequency? Please specify:** Participants randomized to the vegan diet arm were instructed to *avoid all* animal products for the course of the study
3. **Please describe a potential "whole-foods aspect". The dietary intervention explicitly:**
   ☒Favored whole/unprocessed foods
   ☒Restricted: ☒Processed foods ☒Added sugar ☐Added oils
4. **Did the intervention restrict calories or portion sizes?**
   ☒No, *ad-libitum* caloric intake
   ☐Yes, total calorie intake/portion sized restricted
5. **Did the intervention restrict sodium intake?**
   ☒No, *ad-libitum* sodium intake
   ☐Yes, daily sodium intake was restricted
6. **Did the intervention restrict intake of "fatty" plant-foods?**
   ☒No, *ad-libitum* intake
   ☐Yes, the intervention restricted: ☐nuts and seeds ☐oils ☐avocados
7. **Was there a target for macronutrient distribution?**
   ☒No
   ☐Yes
8. **Were participants asked to take any specific supplements during the dietary intervention?**
   ☒No
   ☐Yes ☐Vitamin B12 ☐Vitamin D ☐Calcium ☐Iodine ☐Other: Specify
9. **Did participants receive any kind of support or supervision during the dietary modification?**
   ☐No
   ☒Yes
      ☒Written instructions
      ☒Classes and group sessions
      ☐Cooking demonstrations
      ☒Other – support from health educators throughout the study

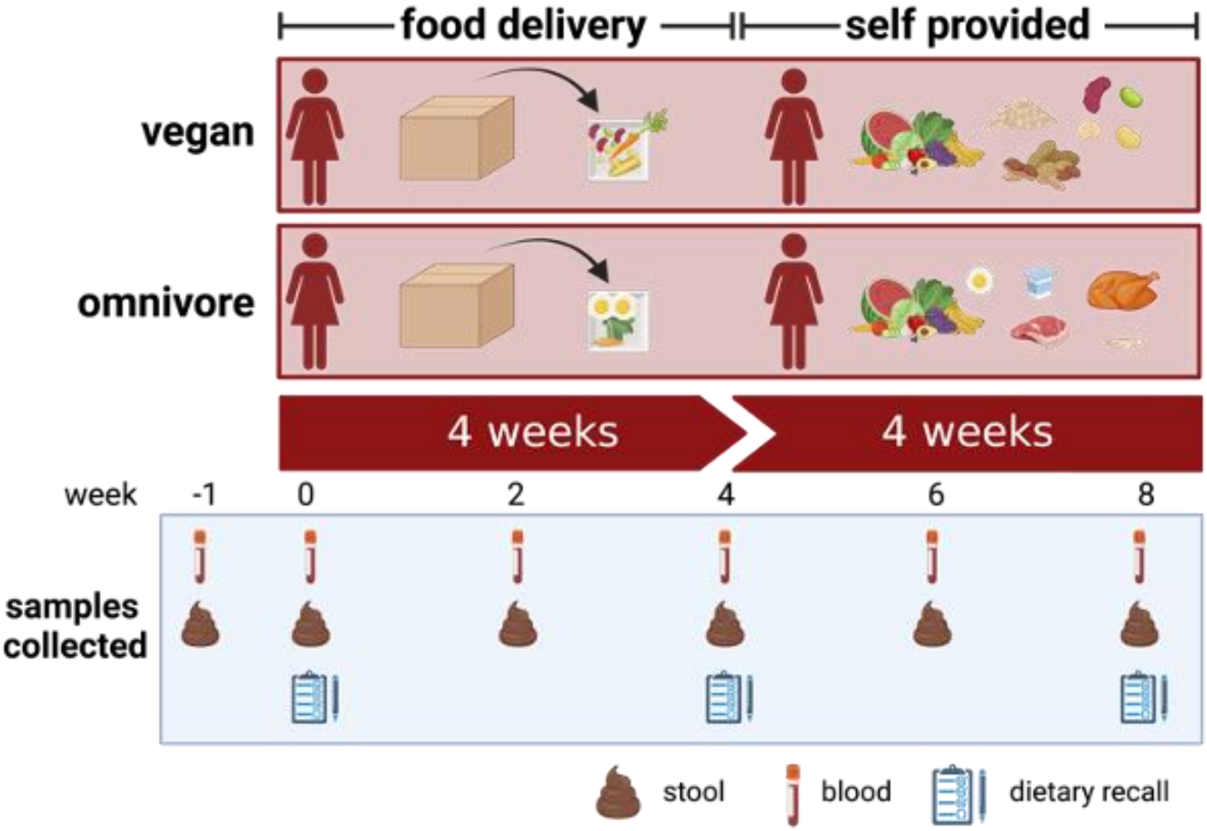

eFigure 1. Twins Nutrition Study (TwiNS) Study Design

© 2023 Landry MJ et al. *JAMA Network Open.*

**eTable 1. Twin Lifestyle Behaviors, n=42 (21 pairs of twins)**	
**Were you and your twin "as alike as two peas in a pod"?**	
As alike as two peas in a pod	29 (69%)
Usual sibling similarity	9 (21%)
Quite different	4 (10%)
**How many years in total have you and your twin lived together since birth?**	22.3 ± 4.6
**Do you and your twin currently live together?**	
Yes	33 (78%)
**At what age did you and your twin first live apart?[1]**	19.7 ± 3.6
**Where has your twin lived, relative to you, in the last 12 months?**	
Within the same suburb/town	26 (62%)
Within the same state	16 (38%)
In a different state	0 (0%)
**When you and your twin are together, how much do you enjoy each other's company?[2]**	4.6 ± 0.6
**When you experience a need for emotional support, how often do you contact your twin for assistance?[2]**	4.2 ± 1.1

Mean ± Standard Deviation or n (%)
[1] Asked only of twins who indicated that they do not currently live with their twin.
[2] Asked on a scale of 1 to 5, lower scores represent lower enjoyment or lower contact.

© 2023 Landry MJ et al. *JAMA Network Open.*

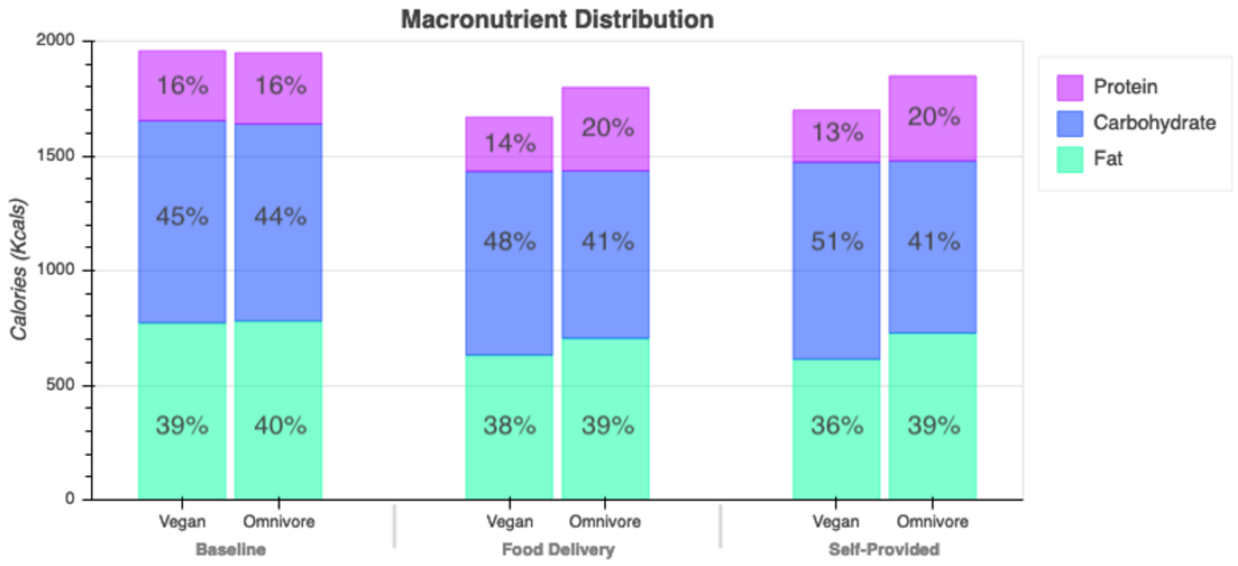

eFigure 2. **Macronutrient Distribution by Phase and Diet.** Data at each timepoint are based on the average of three unannounced 24-hour dietary recalls.

eTable 2. Macronutrient Distribution by Phase and Diet

	**Baseline (n=44)**		**Food Delivery (n=44)**		**Self-Provided (n=43)**	
	**Vegan**	**Omnivore**	**Vegan**	**Omnivore**	**Vegan**	**Omnivore**
Total Energy						
Energy (kcal)	1973 ± 777	1952 ± 851	1628 ± 519	1815 ± 623	1688 ± 653	1862 ± 796
Macronutrient						
Protein (kcal)	304 ± 137	311 ± 154	237 ± 79	364 ± 120	227 ± 93	370 ± 160
Carbohydrate (kcal)	882 ± 379	859 ± 429	802 ± 308	731 ± 266	859 ± 337	751 ± 419
Fat (kcal)	771 ± 383	780 ± 411	631 ± 229	704 ± 361	614 ± 353	727 ± 362

[1]Note: All values are means ± SDs.
[2]Data at each timepoint are based on the average of three unannounced 24-hour dietary recalls.

© 2023 Landry MJ et al. *JAMA Network Open.*

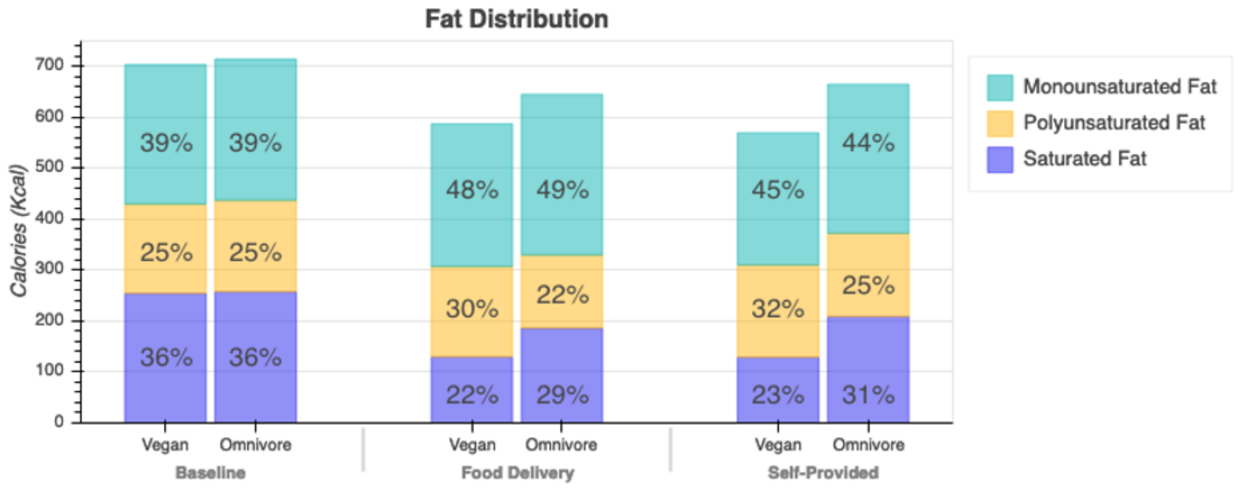

eFigure 3. **Fat Distribution by Phase and Diet.** Data at each timepoint are based on the average of three unannounced 24-hour dietary recalls.

**eTable 3. Fat Distribution by Phase and Diet**						
	**Baseline (n=44)**		**Food Delivery (n=44)**		**Self-Provided (n=43)**	
	**Vegan**	**Omnivore**	**Vegan**	**Omnivore**	**Vegan**	**Omnivore**
Monounsaturated (kcal)	274 ± 147	277 ± 148	280 ± 126	315 ± 217	260 ± 205	292 ± 174
Polyunsaturated (kcal)	175 ± 92	180 ± 118	178 ± 76	144 ± 73	181 ± 106	163 ± 97
Saturated (kcal)	254 ± 147	258 ± 147	130 ± 54	186 ± 100	129 ± 82	209 ± 112

[1]Note: All values are means ± SDs.
[2]Data at each timepoint are based on the average of three unannounced 24-hour dietary recalls.

© 2023 Landry MJ et al. *JAMA Network Open.*

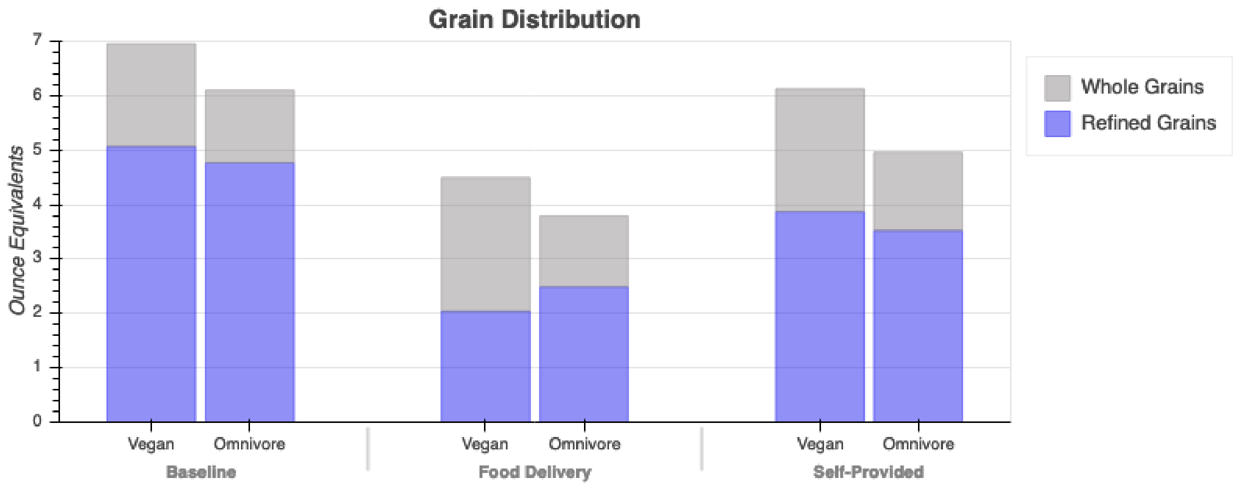

eFigure 4. Grain Distribution by Phase and Diet. Data at each timepoint are based on the average of three unannounced 24-hour dietary recalls.

eTable 4. Grain Distribution by Phase and Diet

	**Baseline (n=44)**		**Food Delivery (n=44)**		**Self-Provided (n=43)**	
	**Vegan**	**Omnivore**	**Vegan**	**Omnivore**	**Vegan**	**Omnivore**
Whole Grains (oz equivalents)	1.89 ± 2.45	1.34 ± 2.45	2.45 ± 2.04	1.30 ± 1.32	2.26 ± 2.18	1.44 ± 1.82
Refined Grains (oz equivalents)	5.07 ± 3.72	4.76 ± 3.59	2.04 ± 2.23	2.49 ± 2.72	3.87 ± 3.34	3.52 ± 4.08

[1]Note: All values are means ± SDs.
[2]Data at each timepoint are based on the average of three unannounced 24-hour dietary recalls.

© 2023 Landry MJ et al. *JAMA Network Open.*

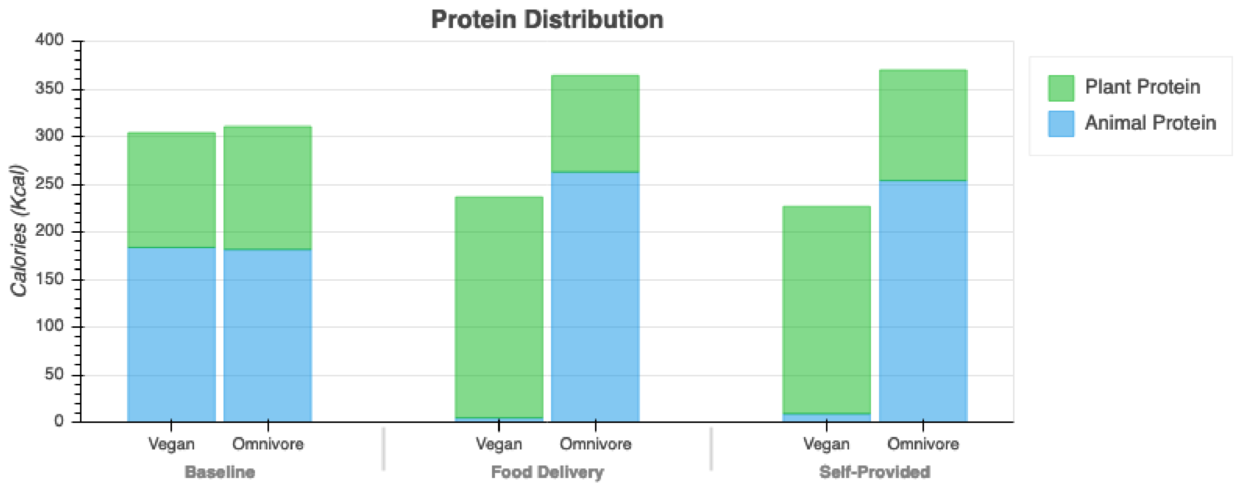

eFigure 5. Protein Distribution by Phase and Diet. Data at each timepoint are based on the average of three unannounced 24-hour dietary recalls.

**eTable 5. Protein Distribution by Phase and Diet**						
	**Baseline (n=44)**		**Food Delivery (n=44)**		**Self-Provided (n=43)**	
	**Vegan**	**Omnivore**	**Vegan**	**Omnivore**	**Vegan**	**Omnivore**
Protein						
Plant (kcals)	120 ± 70	129 ± 85	232 ± 83	102 ± 42	218 ± 96	116 ± 76
Animal (kcal)	184 ± 116	181 ± 110	5 ± 21	263 ± 104	9 ± 26	254 ± 133

[1]Note: All values are means ± SDs.
[2]Data at each timepoint are based on the average of three unannounced 24-hour dietary recalls.

© 2023 Landry MJ et al. *JAMA Network Open.*

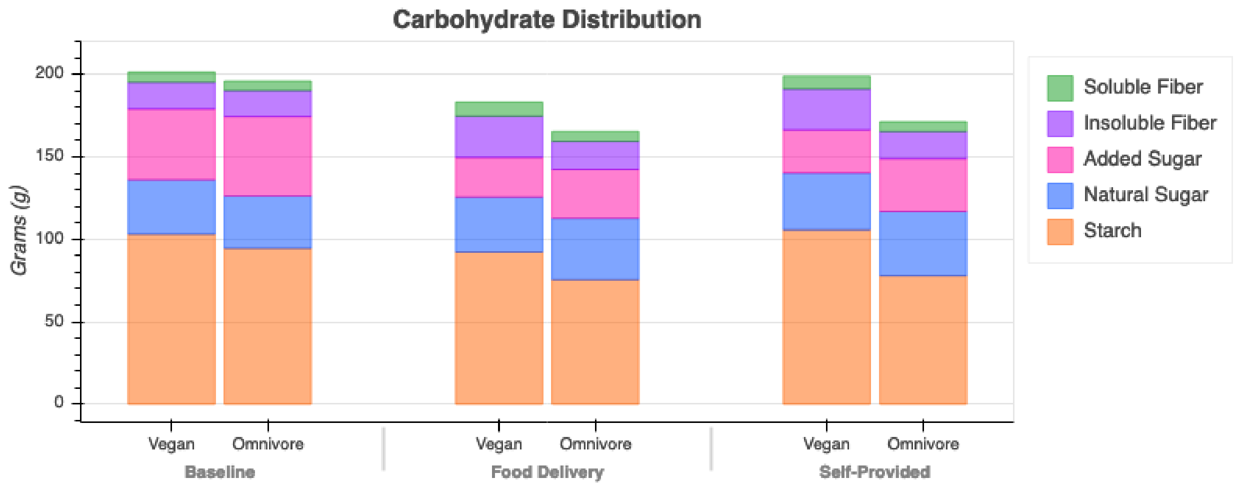

eFigure 6. Carbohydrate Distribution by Phase and Diet. Data at each timepoint are based on the average of three unannounced 24-hour dietary recalls.

**eTable 6. Carbohydrate Distribution by Phase and Diet**						
	**Baseline (n=44)**		**Food Delivery (n=44)**		**Self-Provided (n=43)**	
	**Vegan**	**Omnivore**	**Vegan**	**Omnivore**	**Vegan**	**Omnivore**
Soluble Fiber (g)	6.2 ± 2.9	5.8 ± 3.4	8.5 ± 3.5	6.0 ± 3.1	7.8 ± 3.4	6.0 ± 4.8
Insoluble Fiber (g)	16.1 ± 9.1	15.7 ± 9.3	25.1 ± 9.5	17.0 ± 7.3	24.8 ± 11.2	16.5 ± 10.8
Added Sugar (g)	42.8 ± 36.2	48.2 ± 59.9	24.0 ± 28.6	29.6 ± 30.1	26.2 ± 24.6	32.0 ± 33.5
Natural Sugar (g)	33.0 ± 20.8	31.8 ± 21.7	33.3 ± 19.3	37.5 ± 19.0	34.5 ± 24.7	38.9 ± 25.2
Starch (g)	103.1 ± 64.1	94.5 ± 52.4	92.2 ± 40.6	75.3 ± 36.7	105.7 ± 52.9	77.9 ± 68.0

[1]Note: All values are means ± SDs.
[2]Data at each timepoint are based on the average of three unannounced 24-hour dietary recalls.

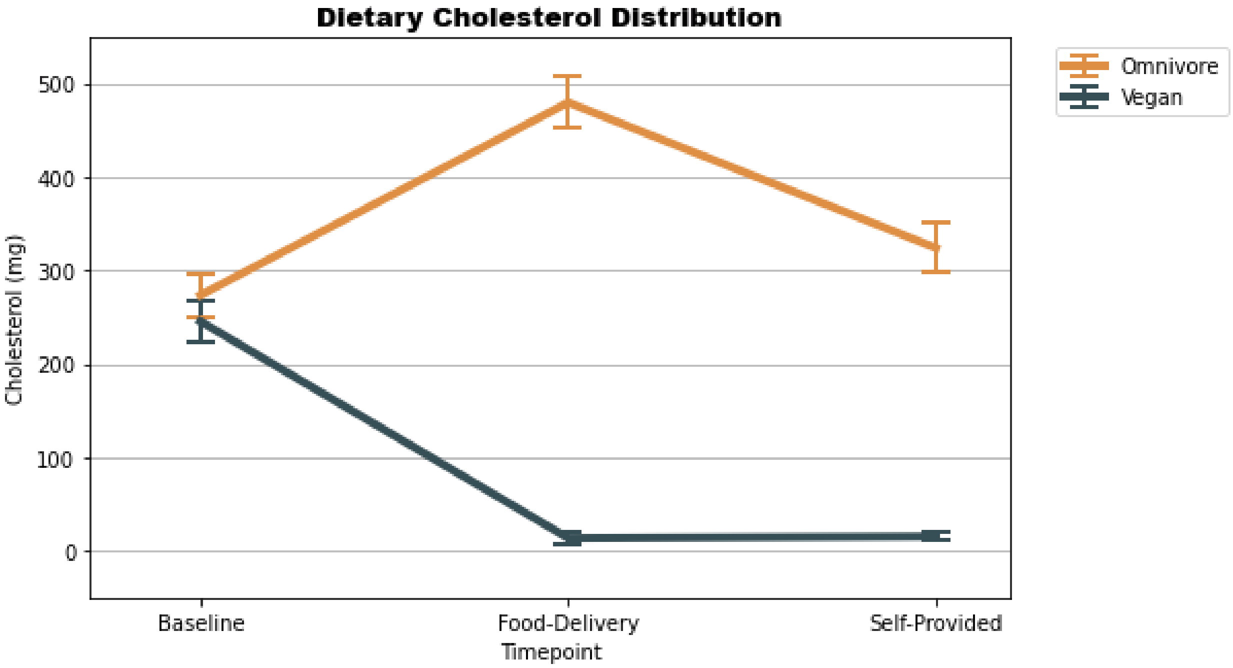

eFigure 7. Dietary Cholesterol Distribution by Phase and Diet (Mean ± SD). Data at each timepoint are based on the average of three unannounced 24-hour dietary recalls.

**SeTable 7: Dietary Cholesterol Distribution by Phase and Diet**						
	**Baseline (n=44)**		**Food Delivery (n=44)**		**Self-Provided (n=43)**	
	**Vegan**	**Omnivore**	**Vegan**	**Omnivore**	**Vegan**	**Omnivore**
Cholesterol (mg)	245.4 ± 173.3	273.5 ± 187.9	13.4 ± 48.1	479.7 ± 222.8	15.2 ± 32.3	324.0 ± 213.8
[1]Note: All values are means ± SDs.<br>[2]Data at each timepoint are based on the average of three unannounced 24-hour dietary recalls.						

© 2023 Landry MJ et al. *JAMA Network Open.*

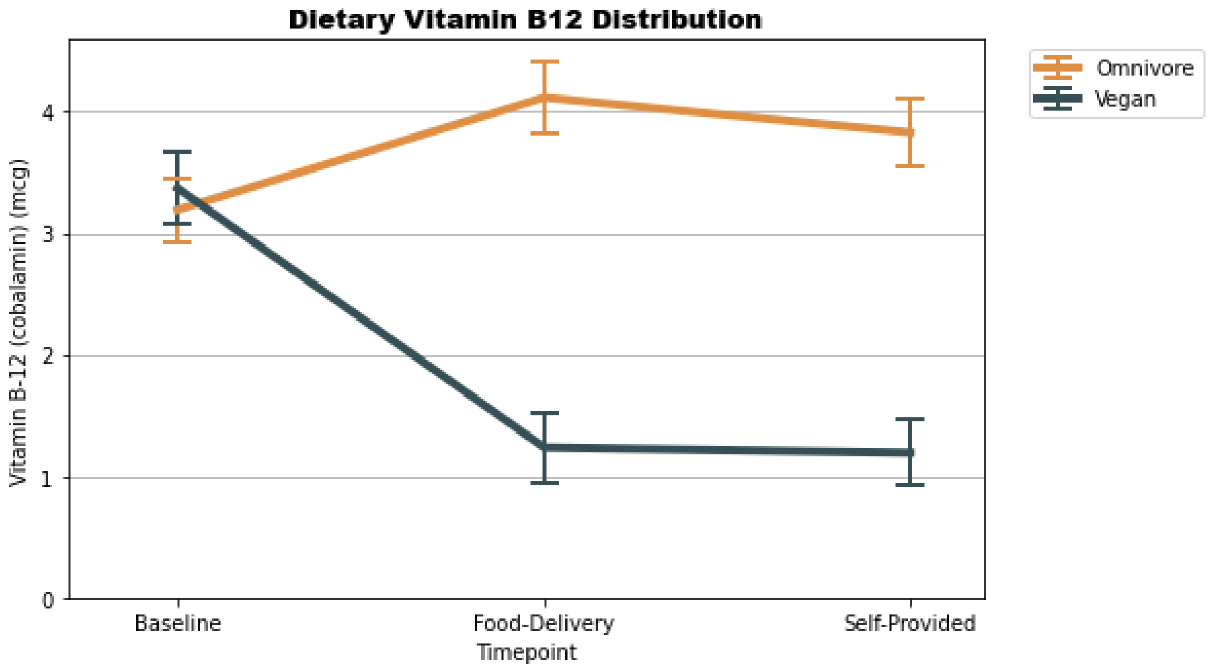

eFigure 8. Dietary Vitamin-B12 Distribution by Phase and Diet (Mean ± SD). Data at each timepoint are based on the average of three unannounced 24-hour dietary recalls.

eTable 8. Dietary Vitamin-B12 Distribution by Phase and Diet

	**Baseline (n=44)**		**Food Delivery (n=44)**		**Self-Provided (n=43)**	
	**Vegan**	**Omnivore**	**Vegan**	**Omnivore**	**Vegan**	**Omnivore**
Vitamin B-12 (mcg)	3.37 ± 2.39	3.19 ± 2.06	1.24 ± 2.31	4.12 ± 2.44	1.19 ± 2.11	3.83 ± 2.24

[1]Note: All values are means ± SDs.
[2]Data at each timepoint are based on the average of three unannounced 24-hour dietary recalls.

© 2023 Landry MJ et al. *JAMA Network Open.*

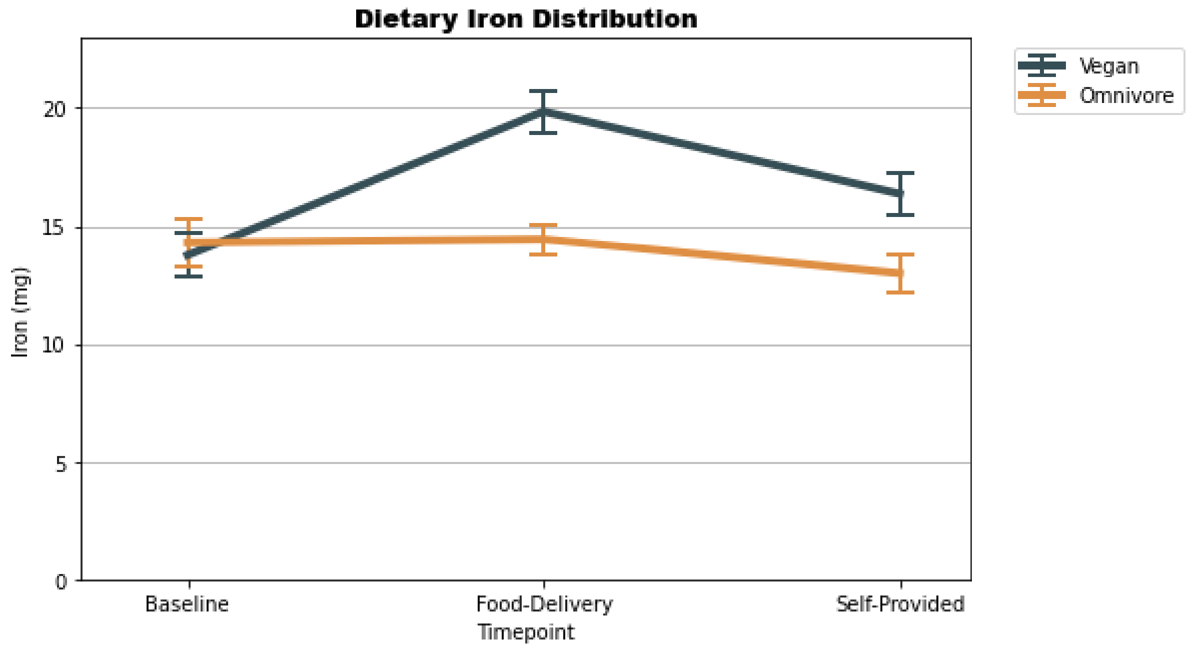

eFigure 9. **Dietary Iron Distribution by Phase and Diet (Mean ± SD)**

**eTable 9. Dietary Iron Distribution by Phase and Diet**						
	**Baseline (n=44)**		**Food Delivery (n=44)**		**Self-Provided (n=43)**	
	**Vegan**	**Omnivore**	**Vegan**	**Omnivore**	**Vegan**	**Omnivore**
Iron (mg)	13.76 ± 7.67	14.29 ± 8.15	19.85 ± 7.15	14.44 ± 5.27	16.37 ± 7.22	12.98 ± 6.33
[1]Note: All values are means ± SDs.<br>[2]Data at each timepoint are based on the average of three unannounced 24-hour dietary recalls.						

© 2023 Landry MJ et al. *JAMA Network Open.*

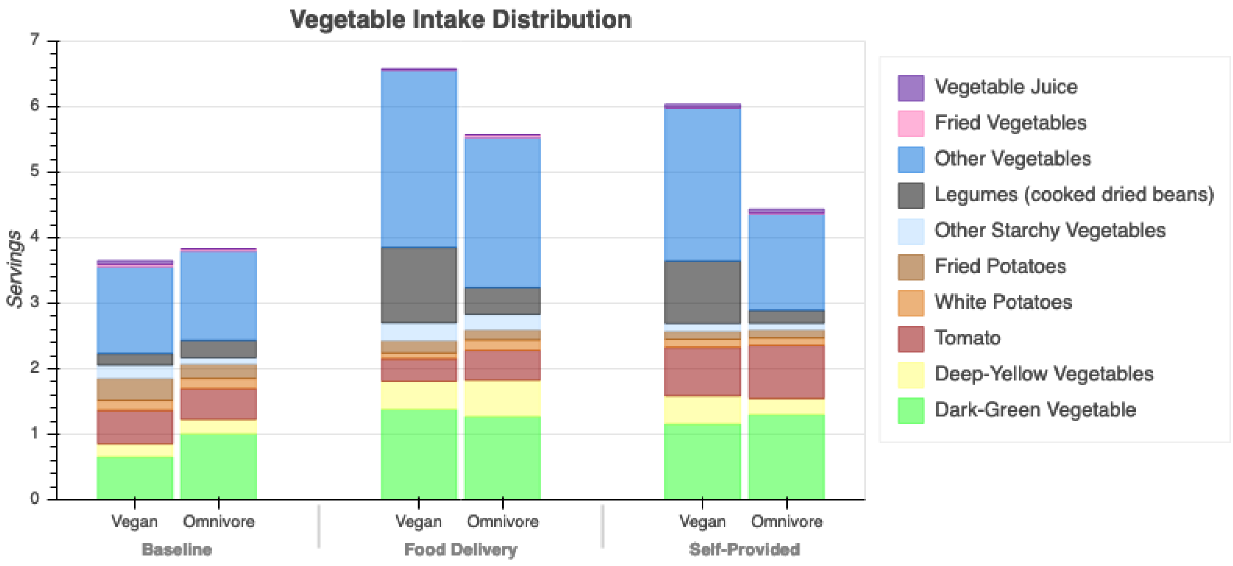

eFigure 10. **Vegetable Servings Distribution by Phase and Diet.** Servings are based on the Nutrition Coordinating Center Food Group Serving Count System. Vegetable servings are defined per the *2000 Dietary Guidelines for Americans* as 1 cup of raw leafy vegetables or ½ cup of other cooked or raw vegetables. When multiple forms of a food are available for a given food, the most common form is selected to represent the serving weight for the food (e.g., chopped, sliced, and grated). Data at each timepoint are based on the average of three unannounced 24-hour dietary recalls.

© 2023 Landry MJ et al. *JAMA Network Open.*

**eTable 10: Vegetable Servings Intake Distribution**						
	**Baseline (n=44)**		**Food Delivery (n=44)**		**Self-Provided (n=43)**	
	**Vegan**	**Omnivore**	**Vegan**	**Omnivore**	**Vegan**	**Omnivore**
Vegetable Juice	0.06 ± 0.49	0.00 ± 0.00	0.02 ± 0.13	0.00 ± 0.01	0.06 ± 0.25	0.06 ± 0.31
Fried Vegetables	0.04 ± 0.35	0.04 ± 0.31	0.01 ± 0.06	0.04 ± 0.25	0.01 ± 0.04	0.02 ± 0.10
Other Vegetables	1.32 ± 1.74	1.36 ± 1.68	2.70 ± 1.80	2.29 ± 1.40	2.33 ± 3.48	1.46 ± 1.61
Legumes (Cooked dried beans)	0.18 ± 0.36	0.27 ± 0.57	1.15 ± 1.01	0.41 ± 0.70	0.95 ± 1.06	0.20 ± 0.39
Other Starchy Vegetables	0.21 ± 0.45	0.09 ± 0.28	0.28 ± 0.31	0.24 ± 0.30	0.13 ± 0.25	0.11 ± 0.22
Fried Potatoes	0.33 ± 0.84	0.22 ± 0.58	0.19 ± 0.54	0.15 ± 0.57	0.12 ± 0.41	0.12 ± 0.38
White Potatoes	0.16 ± 0.55	0.16 ± 0.53	0.09 ± 0.21	0.16 ± 0.42	0.13 ± 0.33	0.12 ± 0.36
Tomato	0.51 ± 0.66	0.47 ± 0.66	0.34 ± 0.46	0.46 ± 0.57	0.74 ± 0.92	0.81 ± 1.43
Deep-Yellow Vegetables	0.20 ± 0.50	0.22 ± 0.63	0.43 ± 0.58	0.55 ± 0.70	0.43 ± 0.77	0.24 ± 0.52
Dark-green Vegetables	0.66 ± 0.99	1.00 ± 1.33	1.38 ± 1.34	1.27 ± 1.31	1.16 ± 1.56	1.30 ± 1.64

[1]Note: All values are means ± SDs.
[2]Data at each timepoint are based on the average of three unannounced 24-hour dietary recalls.

© 2023 Landry MJ et al. *JAMA Network Open.*

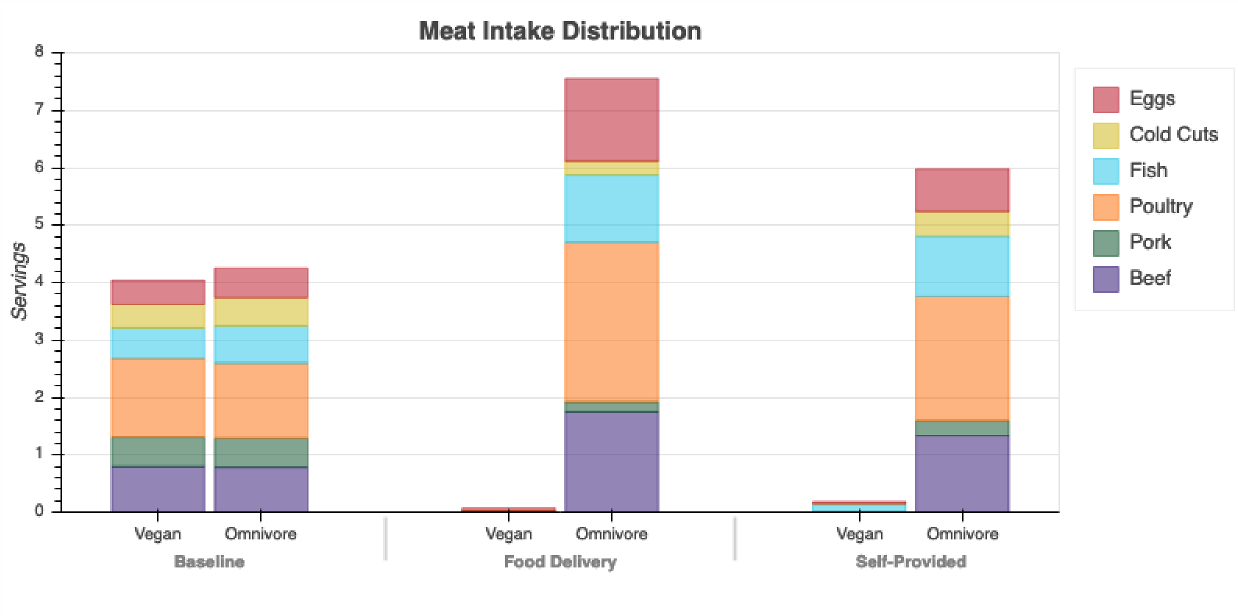

eFigure 11. **Animal-Based Protein Distribution by Phase and Diet.** Servings are based on the Nutrition Coordinating Center Food Group Serving Count System. Servings are generally based on the *2000 Dietary Guidelines for Americans* and are defined in terms of 1-ounce equivalents. One ounce is used for cooked meat, fish, or poultry and 1 ounce-equivalent is used for 1 egg. Data at each timepoint are based on the average of three unannounced 24-hour dietary recalls.

eTable 11. Animal-Based Protein Distribution by Phase and Diet.

	**Baseline (n=44)**		**Food Delivery (n=44)**		**Self-Provided (n=43)**	
	**Vegan**	**Omnivore**	**Vegan**	**Omnivore**	**Vegan**	**Omnivore**
Eggs	0.42 ± 0.69	0.52 ± 0.84	0.05 ± 0.25	1.44 ± 1.06	0.03 ± 0.09	0.74 ± 1.00
Cold Cuts	0.41 ± 0.9	0.49 ± 0.87	0.00 ± 0.00	0.24 ± 0.81	0.00 ± 0.00	0.43 ± 1.09
Fish	0.54 ± 1.51	0.64 ± 1.73	0.01 ± 0.04	1.18 ± 2.16	0.14 ± 0.62	1.04 ± 2.00
Poultry	1.37 ± 2.19	1.31 ± 1.96	0.00 ± 0.00	2.78 ± 2.36	0.01 ± 0.08	2.16 ± 2.70
Pork	0.51± 1.08	0.50 ± 1.56	0.00 ± 0.00	0.17 ± 0.61	0.00 ± 0.00	0.26 ± 0.90
Beef	0.79 ± 1.45	0.78 ± 1.65	0.02 ± 0.15	1.75 ± 2.32	0.00 ± 0.00	1.33 ± 2.18

[1]Note: All values are means ± SDs.
[2]Data at each timepoint are based on the average of three unannounced 24-hour dietary recalls.

© 2023 Landry MJ et al. *JAMA Network Open.*

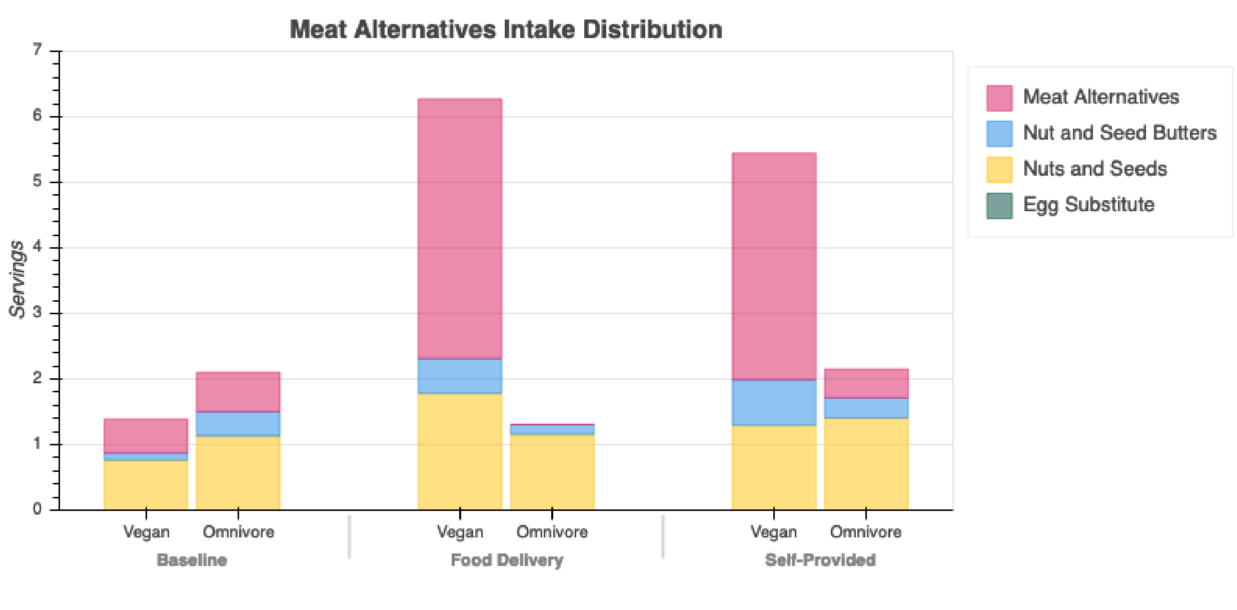

eFigure 12. **Meat Alternatives Distribution by Phase and Diet.** Servings are based on the Nutrition Coordinating Center Food Group Serving Count System. Servings are generally based on the *2000 Dietary Guidelines for Americans* and are defined in terms of 1-ounce equivalents (e.g., 1 tablespoon of peanut butter or ½ ounce of nuts or seeds). Meat alternatives include tofu, tempeh, soy nuts, veggie burgers, etc. Data at each timepoint are based on the average of three unannounced 24-hour dietary recalls.

eTable 12. Meat Alternatives Servings Intake Distribution by Phase and Diet.

	**Baseline (n=44)**		**Food Delivery (n=44)**		**Self-Provided (n=43)**	
	**Vegan**	**Omnivore**	**Vegan**	**Omnivore**	**Vegan**	**Omnivore**
Meat Alternatives	0.52 ± 1.38	0.60 ± 2.64	3.96 ± 3.63	0.00 ± 0.00	3.46 ± 4.32	0.43 ± 1.43
Nut and Seed Butters	0.11 ± 0.31	0.37 ± 0.92	0.54 ± 1.09	0.15 ± 0.51	0.69 ± 1.36	0.31 ± 0.83
Nuts and Seeds	0.77 ± 1.84	1.13 ± 2.20	1.78 ± 2.80	1.16 ± 2.55	1.30 ± 2.21	1.41 ± 2.73
Egg Substitute	0.00 ± 0.00	0.00 ± 0.00	0.00 ± 0.00	0.00 ± 0.00	0.00 ± 0.00	0.00 ± 0.00

[1]Note: All values are means ± SDs.
[2]Data at each timepoint are based on the average of three unannounced 24-hour dietary recalls.

© 2023 Landry MJ et al. *JAMA Network Open.*

eTable 13. Diet Satisfaction (D-Sat28Ó) of Participants by Diet Assignment and Phase.						
	Vegan (n=20)			Omnivore (n=20)		
**Scales**	**Baseline**	**Week 4**	**Week 8**	**Baseline**	**Week 4**	**Week 8**
Healthy Lifestyle	3.7 ± 0.6	4.1 ± 0.4	4.0 ± 0.6	3.5 ± 0.7 /	4.0 ± 0.5	3.9 ± 0.6
Eating Out	4.1 ± 0.8	2.8 ± 1.0	2.6 ± 1.0	4.2 ± 0.8	4.0 ± 0.9	4.2 ± 0.9
Cost	3.0 ± 0.7	3.0 ± 0.8	3.0 ± 0.9	3.1 ± 0.8	3.1 ± 0.7	3.1 ± 0.6
Preoccupation with Food	3.3 ± 0.7	2.9 ± 0.8	2.9 ± 0.9	3.1 ± 0.8	3.2 ± 0.8	3.3 ± 0.7
Planning and Preparation	3.4 ± 0.8	3.0 ± 0.7	2.7 ± 0.9	3.4 ± 0.7	3.4 ± 0.9	3.3 ± 0.9
**Total Diet Satisfaction**	3.5 ± 0.4	3.1 ± 0.5	3.0 ± 0.5	3.5 ± 0.5	3.5 ± 0.6	3.6 ± 0.5
Data are presented as mean ± standard deviation. Scales are measured using five responses ranging from 'Strongly Disagree' to 'Strongly Agree', which are scored from 1 to 5. Select items are reverse scored, so that higher scores indicate greater diet satisfaction. Item scores are averaged to provide five scale scores.						

**eTable 14. Self-Efficacy to Plan, Shop, Cook, and Choose Meals by Diet Assignment and Phase**						
	Vegan (n=20)			Omnivore (n=20)		
**Variable**	**Baseline**	**Week 4**	**Week 8**	**Baseline**	**Week 4**	**Week 8**
To plan meals that follow eating pattern recommendations	4.2 ± 0.9	4.4 ± 0.6	4.3 ± 0.7	3.9 ± 1.1	4.4 ± 1.1	4.4 ± 0.5
To shop and select appropriate foods that follow eating pattern recommendations	4.1 ± 0.9	4.5 ± 0.5	4.4 ± 0.8	4.0 ± 0.9	4.4 ± 1.0	4.4 ± 0.5
To cook meals that follow eating pattern recommendations	4.0 ± 1.0	4.4 ± 0.6	4.3 ± 0.9	3.8 ± 1.1	4.4 ± 1.1	4.4 ± 0.6
To choose foods at restaurants that follow eating pattern recommendations	4.2 ± 0.8	4.4 ± 0.5	4.0 ± 0.9	4.0 ± 1.0	4.4 ± 1.0	4.4 ± 0.6
Data are presented as mean ± standard deviation. Participants asked to self-rate self-efficacy on a scale of 1 to 5, with 1 being strongly disagree, 3 being neither agree nor disagree, and 5 being strongly agree.						

**eTable 15. Diet Preferences of Participants by Diet Assignment**			
	Overall (n=42)	Vegan (n=21)	Omnivore (n=21)
**I found it easier to adhere to dietary recommendations for my assigned eating pattern when**			
I was receiving pre-prepared meals	21 (50.0)	11 (52.4)	10 (47.6)
I was preparing meals on my own	21 (50.0)	10 (47.6)	11 (52.4)
**After this study, I plan to**			
Continue to closely follow all recommendations for my eating pattern	7 (16.7)	1 (4.8)	6 (28.6)
Continue to follow some but not all recommendations for my eating pattern	33 (78.6)	19 (90.5)	14 (66.7)
Return to my original eating pattern before starting the study	2 (4.8)	1 (4.8)	1 (4.8)
Data are presented as n (%)			

© 2023 Landry MJ et al. *JAMA Network Open.*

**eTable 16. Perceptions of Delivered, Pre-Prepared Meals by Diet Assignment**			
How do you rate the...	Overall (n=40)	Vegan (n=20)	Omnivore (n=20)
**Variety to choose the meals you wanted to eat.**			
Poor	10 (25.0)	7 (35.0)	3 (15.0)
Fair	9 (22.5)	4 (20.0)	5 (25.0)
Neutral	7 (17.5)	4 (20.0)	3 (15.0)
Good	10 (25.0)	5 (25.0)	5 (25.0)
Excellent	4 (10.0)	0 (0.0)	4 (20.0)
**Overall quality of meals provided.**			
Poor	8 (20.0)	3 (15.0)	5 (25.0)
Fair	11 (27.5)	6 (30.0)	5 (25.0)
Neutral	3 (7.5)	3 (15.0)	0 (0.0)
Good	13 (32.5)	6 (30.0)	7 (35.0)
Excellent	5 (12.5)	2 (10.0)	3 (15.0)
**Visual appeal of meals.**			
Poor	11 (27.5)	4 (20.0)	7 (35.0)
Fair	9 (22.5)	6 (30.0)	3 (15.0)
Neutral	6 (15.0)	3 (15.0)	3 (15.0)
Good	11 (27.5)	6 (30.0)	5 (25.0)
Excellent	3 (7.5)	1 (5.0)	2 (10.0)
**Cultural appropriateness of meals.**			
Poor	5 (12.8)	2 (10.5)	3 (15.0)
Fair	4 (10.3)	3 (15.8)	1 (5.0)
Neutral	11 (28.2)	6 (31.6)	5 (25.0)
Good	12 (30.8)	5 (26.3)	7 (35.0)
Excellent	7 (17.9)	3 (15.8)	4 (20.0)
**Amount of food provided.**			
Poor	0 (0.0)	0 (0.0)	0 (0.0)
Fair	8 (20.0)	5 (25.0)	3 (15.0)
Neutral	6 (15.0)	4 (20.0)	2 (10.0)
Good	18 (45.0)	8 (40.0)	10 (50.0)
Excellent	8 (20.0)	3 (15.0)	5 (25.0)
Data are presented as n (%)			

© 2023 Landry MJ et al. *JAMA Network Open.*

eTable 17. Barriers to Adherence to Study Eating Patterns by Diet Assignment			
**Which of the following barriers have you experienced within the last 4 weeks regarding your eating pattern?[1]**	Overall (n=40)	Vegan (n=20)	Omnivore (n=20)
Busy lifestyle	27 (61.4)	14 (63.6)	13 (59.1)
Price of foods required to follow my eating pattern	3 (6.8)	2 (9.1)	1 (4.5)
Willpower or self-control	6 (13.6)	1 (4.5)	5 (22.7)
Foods that I do not like	6 (13.6)	4 (18.2)	2 (9.1)
Cooking skills	5 (11.4)	3 (13.6)	2 (9.1)
Time required to prepare meals	20 (45.5)	12 (54.5)	8 (36.4)
Unappealing foods	4 (9.1)	2 (9.1)	2 (9.1)
Foods that spoil or go bad before I use them	11 (25.0)	6 (27.3)	5 (22.7)
Limited cooking equipment	1 (2.3)	1 (4.5)	0 (0.0)
Foods that I am unfamiliar with	3 (6.8)	3 (13.6)	0 (0.0)
Change is too big from prior diet	3 (6.8)	3 (13.6)	0 (0.0)
Does not satisfy hunger	5 (11.4)	4 (18.2)	1 (4.5)
I experience no barriers to adhering to study dietary recommendations	8 (18.2)	3 (13.6)	5 (22.7)

Data are presented as n (%)
[1]Participants were asked to select all that apply.

© 2023 Landry MJ et al. *JAMA Network Open.*

**eTable 18. Factors that Improve Dietary Adherence and Sustainability by Diet Assignment.**			
Variable	Overall (n=42)	Vegan (n=21)	Omnivore (n=21)
**Meeting regularly with the health educator while receiving delivered, pre-prepared meals helped me adhere to eating pattern recommendations.**			
Strongly Disagree	2 (4.8)	1 (4.8)	1 (4.8)
Disagree	2 (4.8)	1 (4.8)	1 (4.8)
Neither Agree or Disagree	9 (21.4)	5 (23.8)	4 (19.0)
Agree	19 (45.2)	9 (42.9)	10 (47.6)
Strongly Agree	10 (23.8)	5 (23.8)	5 (23.8)
**Receiving delivered, pre-prepared meals helped me become more familiar with a healthy [vegan/omnivorous eating pattern].**			
Strongly Disagree	6 (14.3)	2 (9.5)	4 (19.0)
Disagree	4 (9.5)	2 (9.5)	2 (9.5)
Neither Agree or Disagree	8 (19.0)	3 (14.3)	5 (23.8)
Agree	13 (31.0)	10 (47.6)	3 (14.3)
Strongly Agree	11 (26.2)	4 (19.0)	7 (33.3)
**Logging what I ate on Cronometer helped me adhere to eating pattern recommendations.**			
Strongly Disagree	4 (9.5)	1 (4.8)	3 (14.3)
Disagree	4 (9.5)	2 (9.5)	2 (9.5)
Neither Agree or Disagree	12 (28.6)	8 (38.1)	4 (19.0)
Agree	15 (35.7)	7 (33.3)	8 (38.1)
Strongly Agree	7 (16.7)	3 (14.3)	4 (19.0)
Data are presented as n (%)			

© 2023 Landry MJ et al. *JAMA Network Open.*

eTable 19. Average Self-Rated Dietary Adherence by Diet Assignment and Phase (n=40)		
	**Week 4**	**Week 8**
Vegan	4.55 ± 0.69	4.65 ± 0.59
Omnivore	4.35 ± 0.61	4.35 ± 0.49
Data are presented as mean ± standard deviation. Participants asked to self-rate dietary adherence on a scale of 1 to 5, with 1 being not at all, 3 being somewhat, and 5 being following the plan very well.		

© 2023 Landry MJ et al. *JAMA Network Open.*

**eTable 20. Cardiovascular Health Outcomes at the End of 4-weeks and Main Effect Model Estimates, Standard Errors, and 95% Confidence Interval for Primary and Secondary Outcome Analysis**					
**Primary Outcome**	**Vegan (Mean ± SEM)[1]**	**Omnivorous (Mean ± SEM)**	**Estimate[2]**	**Standard Error[2]**	**95% Confidence Interval[2]**
LDL-C (mg/dL)	91.3 ± 6.5	122.4 ± 7.8	-23.1	5.4	- 33.7, -12.5
**Secondary Outcomes**					
Fasting					
HDL-C (mg/dL)	55.2 ± 3.2	61.9 ± 3.5	-2.8	2.1	-6.8, 1.3
Triglycerides (mg/dL)	99.2 ± 11.5	115.1 ± 17.2	-12.0	17.4	-45.7, 21.8
B-12 (pg/mL)	520.8 ± 70.3	497.6 ± 42.3	-68.9	56.7	-180.4, 41.7
TMAO (uM)	3.1 ± 0.4	4.3 ± 0.5	-1.3	2.7	-6.6, 4.0
Glucose (mg/dL)	88.8 ± 1.8	92.1 ± 1.9	-1.7	2.4	-6.3, 3.0
Insulin (uIU/mL)	11.4 ± 1.0	14.7 ± 2.7	-3.1	2.0	-7.0, 0.8
Weight (kg)	71.1 ± 2.8	71.3 ± 2.7	-1.0	0.5	-2.0, 0.1

[1] Means and standard error of the mean (SEM) are unadjusted.
[2] For primary and secondary outcomes, model effect estimates, standard errors, and 95% confidence interval are presented are presented and include fixed effects for diet and time (baseline as reference) and an interaction effect for diet (omnivore as reference) by time, and a random effect for twin pair, to account for the correlation between identical twins (i.e., random intercept allowed intercept to vary for each twin pair).
Abbreviations: SEM, Standard Error of the Mean; Low Density Lipoprotein Cholesterol; HDL-C, High Density Lipoprotein Cholesterol; TMAO, Trimethylamine N-Oxide

© 2023 Landry MJ et al. *JAMA Network Open.*

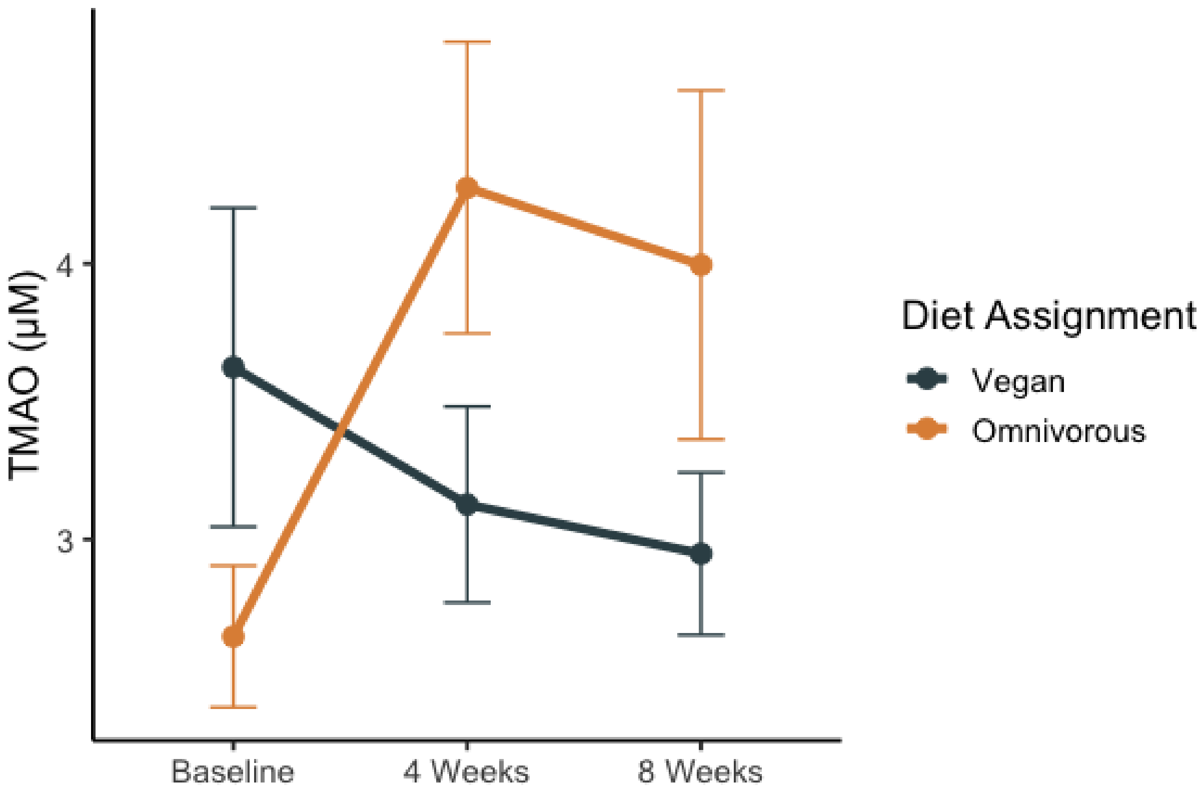

eFigure 13. Sensitivity analysis of the change in trimethylamine N-oxide (TMAO) with three outliers removed, (Mean ± SE).

© 2023 Landry MJ et al. *JAMA Network Open.*

eTable 21. Paired T-Tests of Cardiovascular Health Outcomes at the End of 8-weeks			
	**p-value**	**95% Confidence Interval**	**Sample Estimate**
LDL-C (mg/dL)	0.007	-29.79, -5.41	-17.6
HDL (mg/dL)	0.064	-9.40, 0.30	-4.55
Triglycerides (mg/dL)	0.768	-31.93, 23.93	-4
B12 (pg/mL)	0.208	-233.03, 54.13	-89.45
TMAO (uM)	0.54	-9.05, 4.91	-2.07
Glucose (mg/dL)	0.805	-4.05, 5.15	0.55
Insulin (uIU/mL)	0.133	-6.11, 0.87	-2.62
Weight (kg)	0.002	-3.10, -0.83	-1.96
Abbreviations: Low Density Lipoprotein Cholesterol; HDL-C, High Density Lipoprotein Cholesterol; TMAO, Trimethylamine N-Oxide			

© 2023 Landry MJ et al. *JAMA Network Open.*

eTable 22. Unpaired T-Tests of Cardiovascular Health Outcomes at the End of 8-weeks			
	**p-value**	**95% Confidence Interval**	**Sample Estimate (Vegan, Omnivore)**
LDL-C (mg/dL)	0.004	-29.20, -6.00	-16.85, 0.75
HDL (mg/dL)	0.079	-9.66, 0.56	-4.35, 0.20
Triglycerides (mg/dL)	0.806	-37.22, 29.21	-10.9, -6.9
B12 (pg/mL)	0.211	-233.59, 54.69	-96.15, -6.70
TMAO (uM)	0.52	-8.55, 4.41	-2.12, -0.05
Glucose (mg/dL)	0.825	-4.48, 5.58	0.15, -0.40
Insulin (uIU/mL)	0.054	-5.29, 0.05	-2.10, 0.53
Weight (kg)	0.009	-3.40, -0.53	-2.34, -0.37
Abbreviations: Low Density Lipoprotein Cholesterol; HDL-C, High Density Lipoprotein Cholesterol; TMAO, Trimethylamine N-Oxide			

eTable 23. Average Macronutrient and Micronutrient Composition of Trifecta Food Delivery Meals by Meal Type and Diet Assignment[1]				
**Nutrient**	**Breakfast Meals**		**Entrée Meals**	
	**Vegan**	**Omnivore**	**Vegan**	**Omnivore**
Energy (kcal)	280	310	390	433
Total Fat (g)	7	19	16	16
Total Carbohydrate (g)	47	13	48	40
Total Protein (g)	10	23	20	33
Total Saturated Fatty Acids (g)	1	4	3	4
Total Monounsaturated Fatty Acids (g)	3	9	7	8
Total Polyunsaturated Fatty Acids (g)	2	3	5	3
Total Dietary Fiber (g)	7	3	11	6
Sodium (mg)	108	532	485	537
Total Sugars (g)	11	5	8	7
Added Sugars (g)	4	0	1	2

[1]Participants received one breakfast item and two entrée items per day and were expected to supplement with additional food to meet individual needs as coached by health educators.

Data Sharing Statement

Data available: No, but will be made available upon request.

Additional Information Explanation for why data not available: Participant data was collected and used for the present study. A publication of the dataset in public repositories is not possible for data protection reasons, but the analytical dataset will be made available by the corresponding author upon request.

© 2023 Landry MJ et al. *JAMA Network Open.*

eReferences

1. Gardner CD, Crimarco A, Landry MJ, Fielding-Singh P. Nutrition Study Design Issues—Important Issues for Interpretation. *Am J Health Promot*. 2020-11-01 2020;34(8):951-954. doi:10.1177/0890117120960580d
2. Storz MA. What makes a plant-based diet? A review of current concepts and proposal for a standardized plant-based dietary intervention checklist. *Eur J Clin Nutr*. 2022;76(6):789-800.
3. Miller WG, Myers GL, Sakurabayashi I, et al. Seven direct methods for measuring HDL and LDL cholesterol compared with ultracentrifugation reference measurement procedures. *Clin Chem*. 2010;56(6):977-986.
4. Rambaldi DC, Reschiglian P, Zattoni A, Johann C. Enzymatic determination of cholesterol and triglycerides in serum lipoprotein profiles by asymmetrical flow field-flow fractionation with on-line, dual detection. *Anal Chim Acta*. 2009;654(1):64-70.
5. Bystrom C, Sheng S, Zhang K, Caulfield M, Clarke NJ, Reitz R. Clinical utility of insulin-like growth factor 1 and 2; determination by high resolution mass spectrometry. *PLoS One*. 2012;7(9):e43457.
6. Peterson JI, Young DS. Evaluation of the hexokinase/glucose-6-phosphate dehydrogenase method of determination of glucose in urine. *Anal Biochem*. 1968;23(2):301-316.
7. Friedewald WT, Levy RI, Fredrickson DS. Estimation of the concentration of low-density lipoprotein cholesterol in plasma, without use of the preparative ultracentrifuge. *Clin Chem*. 1972;18(6):499-502.
8. Tang WW, Wang Z, Levison BS, et al. Intestinal microbial metabolism of phosphatidylcholine and cardiovascular risk. *N Engl J Med*. 2013;368(17):1575-1584.
9. James B, Loken E, Roe L, et al. Validation of the diet satisfaction questionnaire: a new measure of satisfaction with diets for weight management. *Obes Sci Pract*. 2018;4(6):506-514.
10. O'Reilly H, Panizza CE, Lim U, et al. Utility of self-rated adherence for monitoring dietary and physical activity compliance and assessment of participant feedback of the Healthy Diet and Lifestyle Study pilot. *Pilot and Feasibility Studies*. 2021;7(1):1-10.
11. Costabile G, Vetrani C, Bozzetto L, et al. Plasma TMAO increase after healthy diets: results from 2 randomized controlled trials with dietary fish, polyphenols, and whole-grain cereals. *Am J Clin Nutr*. 2021;114(4):1342-1350.
12. Gardner CD, Kiazand A, Alhassan S, et al. Comparison of the Atkins, Zone, Ornish, and LEARN Diets for Change in Weight and Related Risk Factors Among Overweight Premenopausal Women. *JAMA*. 2007;297(9):969. doi:10.1001/jama.297.9.969

© 2023 Landry MJ et al. *JAMA Network Open.*
