# Supplementary material for: Cardiometabolic Effects of Omnivorous vs Vegan Diets in Identical Twins: A Randomized Clinical Trial
Source: JAMA Netw Open. 2023 Nov 30;6(11):e2344457. doi: 10.1001/jamanetworkopen.2023.44457 (PMC10690456; doi:10.1001/jamanetworkopen.2023.44457)
Supplement: Supplement 3. — Data Sharing Statement [file jamanetwopen-e2344457-s003.pdf]

Data Sharing Statement

Landry. Cardiometabolic Effects of Omnivorous vs Vegan Diets in Identical Twins. *JAMA Netw Open*. Published November 30, 2023. doi:10.1001/jamanetworkopen.2023.44457

Data
Data available: Yes
Data types: Deidentified participant data, Data dictionary
How to access data: cgardner@stanford.edu
When available: With publication

Supporting Documents
Document types: None

Additional Information
Who can access the data: Researchers whose proposed use of the data has been approved.
Types of analyses: For any research purpose.
Mechanisms of data availability: With a signed Data Use Agreement.
